# Single-cell transcriptomics unravels the early immune landscape of renal allograft rejection and nominates Ccl3-Ccr5 as a therapeutic target

**DOI:** 10.3389/fimmu.2025.1663251

**Published:** 2025-10-22

**Authors:** Fanchao Wei, Zhaoxiang Wang, Ruochen Qi, Jingliang Zhang, Shichao Han, Changhong Shi, Tong Lu, Zhite Zhao, Zhengxuan Li, Lang Li, Weijun Qin, Shuaijun Ma, Lijun Yang

**Affiliations:** 1 Department of Urology, Xijing Hospital, Air Force Medical University, Xi’an, Shaanxi, China; 2 Division of Cancer Biology, Laboratory Animal Center, Air Force Medical University, Xi’an, Shaanxi, China; 3 Skills Training Center, Xijing Hospital, Air Force Medical University, Xi’an, Shaanxi, China

**Keywords:** kidney transplant, acute rejection, macrophages, ScRNA-seq, Cc3-Ccr5

## Abstract

**Background:**

Acute rejection is a significant cause of impaired graft survival in the early post-transplantation period, and the early-stage immune cell dynamics with local intercellular communication during this process require further elucidation.

**Methods:**

We performed single-cell RNA sequencing (scRNA-seq) on CD45+ immune cells isolated from rat renal allografts during the early phase of acute rejection (days 0, 1, 3, and 7). Using unsupervised clustering, functional enrichment analysis, cellular trajectory inference, and intercellular communication network mapping, we delineated the immune cell dynamics and local communication networks at single-cell resolution. Our findings were subsequently validated through multiplex immunofluorescence and therapeutic intervention experiments.

**Results:**

Macrophages constituted the dominant immune population during acute rejection. Sub-clustering analysis revealed a rapid expansion of the Isg15+Mac subset by post-transplant day 1, which persisted at elevated levels thereafter. Functional enrichment and trajectory inference demonstrated the pro-inflammatory properties of Isg15+Mac, implicating this subset in acute rejection. Cell-cell communication analysis identified Ccl3-Ccr5 ligand-receptor interactions between Isg15+Mac and T cells. Multiplex immunofluorescence confirmed abundance of Isg15+Mac within the allografts. Moreover, the acute rejection after kidney transplantation was alleviated by the FDA-approved Ccr5 blocker Maraviroc.

**Conclusions:**

Our study establishes an in-depth, early-stage immune landscape of renal transplantation, revealed that the Isg15+Mac subset activates T cells via the Ccl3–Ccr5 axis and thereby serves as a critical driver of acute rejection. And indicating that Maraviroc may potentially be a therapeutic candidate for transplant rejection.

## 1 Introduction

Renal transplantation remains the most effective treatment for end-stage renal disease. However, acute rejection remains a significant cause of graft loss within the first year after surgery (1). Within the first year post-transplantation, the incidence of clinical acute rejection ranges from 10% to 15%, while subclinical rejection occurs in 5% to 15% of recipients. Up to 40% of transplant recipients may have subclinical inflammation (borderline changes suggestive of rejection) during the first year after transplantation (2). Furthermore, acute rejection can predispose patients to chronic allograft nephropathy, which is strongly associated with an increased risk of long-term graft failure and mortality (3). T lymphocytes and B lymphocytes have long been established as central players in mediating acute allograft rejection (4, 5), Accumulating evidence reveals pivotal and expanding roles of innate immune cells — macrophages, dendritic cells, and NK cells — during the incipient phase of acute rejection (6–8). These cells are rapidly activated post-transplantation, executing critical functions such as antigen presentation, pro-inflammatory mediator secretion, and priming of adaptive immunity (9). Consequently, comprehensive delineation of early immune dynamics within allografts and their precise contributions to rejection pathogenesis is imperative.

Single-cell RNA sequencing (scRNA-seq) is a powerful tool for profiling gene expression at single-cell resolution and deciphering intercellular signaling networks. Previous studies have demonstrated its distinct advantages in characterizing renal development and modeling diverse kidney pathologies (10–12). At the same time, more and more evidence also shows that scRNA-seq technology can effectively analyze the dynamic cellular landscape after transplantation in acute rejection, and play important roles in these aspects: identifying new cell subtypes, studying cell communication after acute rejection, revealing the mechanisms of acute rejection and potential therapeutic targets (13). In renal transplantation, scRNA-seq provides a powerful new lens into rejection mechanisms. Some studies have revealed M1-like macrophages correlating with the severity of pathological injury in renal allografts (14), scRNA-seq can also unveil the immune landscape in chronically rejected renal allografts and identify distinct fibroblast subpopulations critically involved in chronic rejection pathogenesis (15). Moreover, prior studies leveraging scRNA-seq have defined the immunological signatures of rejecting murine renal allografts at post-transplant days 7 and 15 (16, 17). However, the immune cell dynamics and local intercellular communication networks during the innate immunity-dominated early phase (within 7 days post-transplantation) of renal allograft acute rejection remain incompletely characterized (18, 19), Therefore, further research needs to be carried out at the single-cell level.

In this study, we integrated CD45+ cell sorting with scRNA-seq to delineate the immune cell dynamics in rat renal allografts during early acute rejection (days 1, 3, and 7 post-transplantation). We identified six major cell types, and further analyzed the relevant subsets and immunological characteristics of macrophages and T cells. We discovered that on the one hand macrophages dominated the immune cell population, and on the other hand they rapidly underwent phenotypic transformation after transplantation. Among them, the proportion of Isg15+Mac increased substantially at day 1 post-transplantation. GO enrichment analysis found that this subset mainly played pro-inflammatory function. Multiplex immunofluorescence also confirmed the abundant existence of this subset after transplantation. By further mining cell-cell communication through Cellchat, we found that Isg15+Mac communicated with T cells and mediated the occurrence of acute rejection through Ccl3-Ccr5 ligand-receptor interaction. Finally, using Maraviroc to block the Ccr5 receptor significantly inhibited acute renal transplant rejection. Maraviroc is a highly selective CCR5 antagonist that was FDA-approved in 2007 for the treatment of HIV infection (20). In recent years, this drug has demonstrated potential in the management of autoimmune diseases, pancreatic cancer, colorectal cancer, and graft-versus-host disease (GVHD) (21–23). However, a research gap remains in the field of solid organ transplantation. In the present study, we have, for the first time, repurposed Maraviroc for ultra-early intervention in renal transplant rejection, with promising therapeutic efficacy achieved.

In summary, our single-cell transcriptome data can become a more in-depth and earlier research resource for the mechanisms of acute rejection. And it provides new therapeutic targets for inhibiting rejection.

## 2 Materials and methods

### 2.1 Establishment of rat orthotopic kidney transplantation model

Male Wistar and Sprague-Dawley (SD) rats aged 6–8 weeks (body weight 200–250 g) were obtained from the Animal Experiment Center of the Air Force Medical University. An acute renal allograft rejection model was established by orthotopic transplantation of kidneys from male Wistar rats into SD recipients (24), Wistar→SD pairs were selected for their defined MHC disparity. Both donors and recipients were maintained under anesthesia with Zoletil 50 (0.1–0.12 ml/100g). The recipient’s native left kidney was excised. The left kidney from the Wistar rat was transplanted into the left abdominal cavity of the SD rat, The renal artery and vein of the graft were anastomosed end-to-end with the left renal artery and vein of the SD recipient, respectively. The ureter of the graft was anastomosed to the bladder of the SD rat. All rat experiments were conducted in specific pathogen-free (SPF) facilities in accordance with the guidelines of the Animal Care and Use Committee of Air Force Medical University. Postoperatively, animals were euthanized under Zoletil 50 anesthesia at designated time points (0, 1, 3, and 7 days) for sample collection. The experimental protocol was approved by the Animal Ethics Committee of Air Force Medical University(KY20223099-1).

### 2.2 Single-cell suspension preparation and flow cytometry cell sorting

At each time point, transplanted kidneys were harvested from 3 rats per group. Following euthanasia, kidneys were perfused via the abdominal aorta with ice-cold phosphate-buffered saline (PBS) for blood clearance. The three kidneys per group were pooled, minced into 1–2 mm³ fragments, and digested in collagenase (17018029/17104019, eBioscience™) at 37 °C for 45 min. The digestate was filtered through a 40-μm cell strainer. Erythrocytes were lysed using 1 ml ice-cold RBC Lysis Buffer (C3702, Beyotime) for 5 min, followed by PBS washing to obtain a single-cell suspension. The prepared single-cell suspension was counted and examined by microscopy. Cells were incubated with anti-CD45-APC antibody (17-0461-82, eBioscience™) at 4 °C for 30 min. During the last 5 min of incubation, DAPI (1:1000 dilution, 62248, eBioscience™) was added and mixed. After incubation, cells were washed once with RPMI-1640 medium (12633020, eBioscience™) via centrifugation (300g, 5 min), resuspended, and filtered through a 40-μm cell strainer. The final suspension was transferred to flow cytometry tubes and kept protected from light on ice. Pre-prepared collection tubes containing RPMI-1640 with 10% FBS were loaded into the sorting chamber for CD45^+^ cell sorting. After sorting, the fluidics system was flushed with sterile ultrapure water. A subset of sorted cells was re-analyzed for quality control, and flow cytometry data were recorded. The target cell suspension was transferred from flow tubes to 15-mL centrifuge tubes, pelleted by centrifugation, counted, examined microscopically, and processed for single-cell library preparation.

### 2.3 Single-Cell RNA sequencing

The cell suspension was loaded into Chromium microfluidic chips with 3’ (v2 or v3, depends on project) chemistry and barcoded with a 10× Chromium Controller (10X Genomics). RNA from the barcoded cells was subsequently reverse-transcribed and sequencing libraries constructed with reagents from a Chromium Single Cell 3’ v2(v2 or v3, depends on project) reagent kit (10X Genomics) according to the manufacturer’s instructions. Sequencing was performed with Illumina (Hi-Seq 2000 or Nova-Seq, depends on project) according to the manufacturer’s instructions (Illumina).

### 2.4 Generation of single-cell transcriptomes and quality control

Cell Ranger (v7.1.0, 10x Genomics) was used to align raw FASTQ files to the rat reference genome mRn7 (GCA_015227675.2). Seurat (v5.0.3) was employed for quality control, data preprocessing, and dimensionality reduction. Specimens at each time point (0, 1, 3, 7 days) were derived from transplanted kidneys of 3 rats per group. After obtaining the gene-cell matrices from transplanted and control groups, all matrices were merged. To filter low-quality cells, we applied the following criteria: Cells with >500 genes detected and <5,000 genes; Cells with mitochondrial gene percentage <5%; Genes detected in ≥5 cells with ≥1 feature count.

### 2.5 Analysis of single-cell transcriptomes and cell clustering

The high-quality single cells were re-processed through the full Seurat workflow to generate the final dataset for all downstream analyses. Major cell types were identified using standard Seurat (v5.0.3) clustering at a resolution of 0.6. Cellular clusters were visualized via uniform manifold approximation and projection (UMAP). Cluster-specific marker genes were selected from differentially expressed genes (DEGs) using Seurat’s FindAllMarkers function. Six major cell types in the final dataset were manually annotated based on canonical marker gene sets.Visualizations were generated using Seurat plotting functions: VlnPlot; FeaturePlot; DotPlot; DoHeatmap.

### 2.6 Visualization of differential abundance neighborhoods

We employed the MiloR package (v1.12.0) to detect differential abundance of cell populations across experimental conditions by modeling cell counts within k-nearest neighbor (KNN) graph neighborhoods. To visualize results from differential analysis on neighborhoods, we construct an abstracted graph, where nodes represent neighborhoods and edges represent the number of cells in common among neighborhoods. The size of nodes represents the number of cells in the neighborhood. The position of nodes is determined by the position of the sampled index cell in the single-cell uniform manifold approximation and projection (UMAP), to allow qualitative comparison with the single-cell embedding. Graphical visualization is implemented using the R packages ggplot and ggraph.

### 2.7 Enrichment analysis

Gene Ontology (GO) enrichment analysis was performed using the enrichGO function from the R package clusterProfiler (v4.10.0). KEGG pathway analysis was conducted with the Kyoto Encyclopedia of Genes and Genomes database (KEGG release 110.1). Results were visualized using R packages ggplot2 (v3.5.0) and enrichplot (v1.26.0). Volcano plots generated via ggplot2 displayed significantly upregulated/downregulated genes (|log_2_FC| > 1, adj. p < 0.05).

### 2.8 Sub-clustering analysis

Subclustering analysis of target cells was performed using Seurat (v5.0.3) at a resolution of 0.6. Cellular subclusters were visualized via Uniform Manifold Approximation and Projection (UMAP). Marker genes for each subcluster were identified from differentially expressed genes (DEGs) using Seurat’s FindAllMarkers function (min.pct = 0.25, logfc.threshold = 0.5). Subclusters were manually annotated based on canonical marker genes and known biological functions as described above.

### 2.9 Gene set scoring

Enrichment scores were calculated using Seurat’s AddModuleScore function. This function computes the mean-centered expression of a gene set relative to control gene sets, which represents the average relative expression. Gene sets were curated based on previously published signatures for macrophage lineages and phenotypic functions (**Supplementary Table 2**).

### 2.10 ScRNA-seq trajectory analysis

Pseudotime trajectories for each cell type were constructed from Seurat objects using the R package Monocle 2 (v2.26.0). Pseudotemporally variable genes were identified with Monocle 2’s differential-Gene-Test function (q-value < 0.01). Branch-dependent expression patterns were assessed using Branch Expression Analysis Modeling (BEAM) and visualized via plot-genes-branch-heatmap.

### 2.11 Ligand–receptor interactions

Interaction weights were calculated as the product of ligand fold-change in sender cells and receptor fold-change in receiver cells. Cell-cell communication networks were predicted from single-cell RNA sequencing data using the CellChat library (v1.6.0). Ligand-receptor interactions were inferred with CellChat, considering only ligands and receptors expressed in >5% of cells within corresponding cell types.

### 2.12 Histological staining

Tissues were fixed in 4% paraformaldehyde (PFA), embedded in paraffin wax, sectioned at 5 μm thickness. Paraffin sections were dried overnight on a heating plate at 40 °C. Deparaffinization and rehydration were performed through sequential immersion in xylene and ethanol gradients. Sections were stained with Mayer’s hematoxylin for 3 min, followed by three washes in distilled water. Counterstaining was performed in eosin Y solution for 2 min. Dehydration was achieved through graded ethanol series (70%, 95%, 100%), cleared in xylene for 5 min, and mounted with synthetic resin under cover slips.

### 2.13 Immunohistochemical staining

Renal tissues were dewaxed with xylene. Antigen retrieval was then performed in 0.01 M citrate buffer for 0.5 hours. After incubation in blocking solution containing 0.1% Triton X-100 for 1.5 hours at room temperature, the tissues were incubated overnight with CD3 antibody (1:200 dilution; ab11089, Abcam) and KIM-1 antibody (1:200 dilution; PA5-79345, eBioscience™). After washing away excess antibodies, sections were incubated with secondary antibodies, followed by DAB staining (PR30018, Proteintech), and images were captured.

### 2.14 Multiplex immunofluorescence staining

Immunofluorescence analysis was performed on renal sections. After antigen retrieval, slides were incubated with primary antibodies:Anti-CD3 (1:500 dilution; GB151137, Servicebio); Anti-CD68 (1:200 dilution; GB113109, Servicebio); Anti-ISG15 (1:200 dilution; 15981-1-AP, Proteintech). Following sequential washing, sections were labeled with species-matched secondary antibodies conjugated to Alexa Fluor^®^ dyes (Invitrogen). Nuclei were counterstained with DAPI (1μg/mL, D9542, Sigma-Aldrich). Autofluorescence was quenched with 0.1% Sudan Black B for 30 min. Sections were mounted with anti-fade medium (ProLong Gold, P36930, Thermo Fisher) and imaged using a confocal microscope (Nikon Eclipse C1, Japan) with whole-slide scanning (Pannoramic MIDI, 3DHISTECH).

### 2.15 *In vivo* blockade of the Ccr5 pathway

A Ccr5 inhibitor maraviroc (H-13004, MedChemExpress) was dissolved in 10% DMSO(1000μL)+ 40%PEG300(4000μL)+ 5% Tween-80 (500μL)+ 45% saline(4500μL) and mixed until clear. Kidney transplant rats were intraperitoneally injected daily at a dose of 50 mg/kg starting from 1 day before transplantation and continuing until day 7 after transplantation. In the control group, only 10% DMSO was injected. In addition, venous blood samples were collected from transplanted rats for comprehensive biochemical analysis of serum creatinine and blood urea nitrogen (BUN).

### 2.16 Statistical analysis

Data are expressed as the mean ± standard deviation (SD) and were analyzed by using GraphPad Prism 10.2 software. Two-tailed unpaired Student’s t-test was used for comparisons between two groups. One-way ANOVA was used for comparisons among multiple groups. The P value for graft survival was determined by the Mann–Whitney test. The significance level was set at P < 0.05.

## 3 Results

### 3.1 Progression of acute allograft rejection in rat renal transplantation from day 1 to 7 postoperation

**Figure 1:**
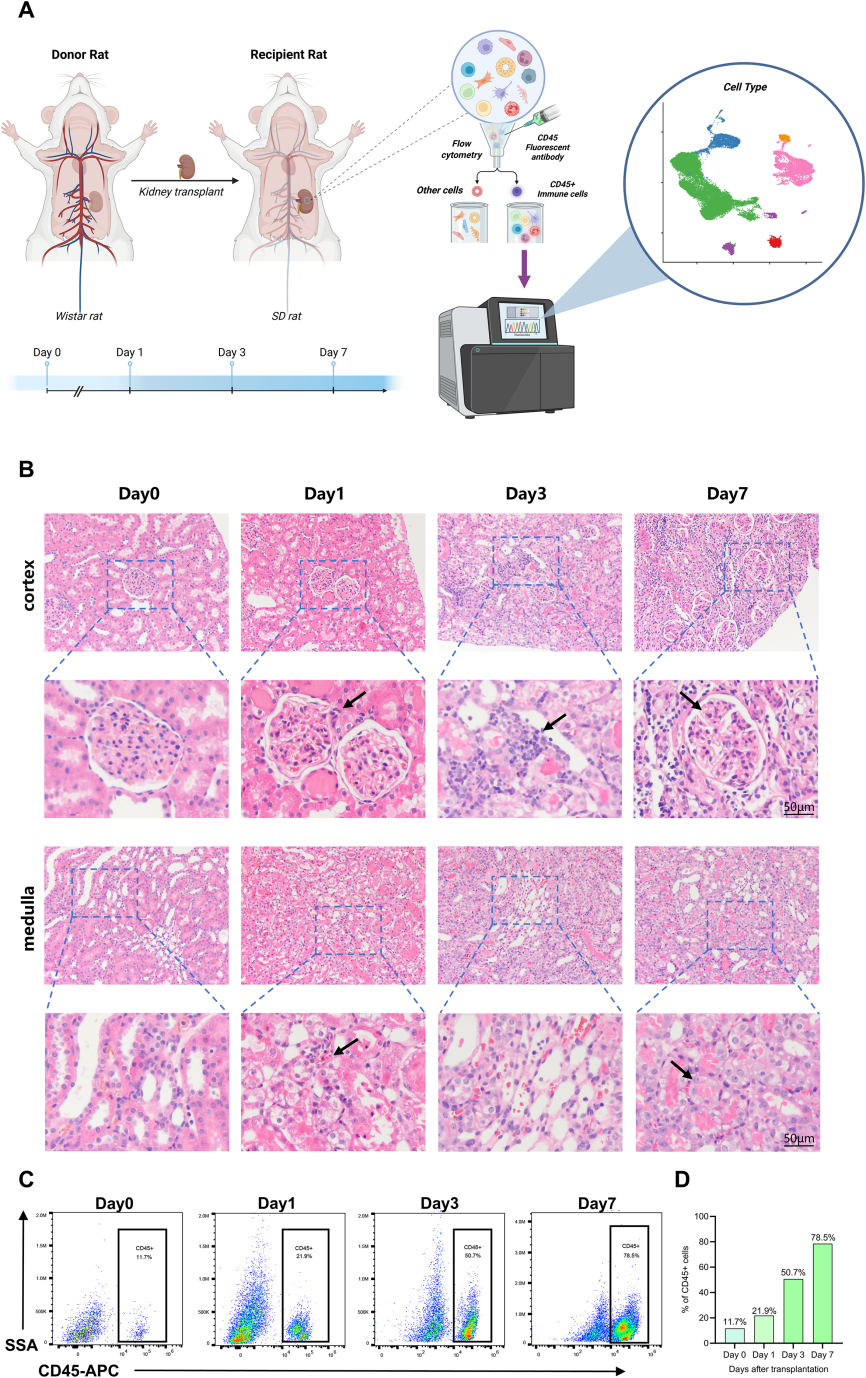
Key histopathological and cellular alterations in renal allografts post-transplantation. **(A)** Experimental workflow. An acute rejection model was induced via renal transplantation in rats. Allograft samples were collected at postoperative days (POD) 0, 1, 3, and 7 (*n=3 per timepoint*), with CD45^+^ immune cells isolated by FACS for single-cell suspension preparation and subsequent 10x Genomics single-cell transcriptome sequencing. **(B)** Representative H&E-stained sections at each time point (Scale bars=50 μm). Progressive histopathological manifestations of acute rejection were observed, including hydropic degeneration of tubular epithelial cells, expanding interstitial inflammatory cell infiltration, and the development of glomerulitis and tubulitis. **(C)** Temporal dynamics of graft-infiltrating CD45^+^ immune cell proportions post-transplantation.

To delineate the immune reconstitution of renal allografts and uncover early mechanisms of acute rejection, we established an allogeneic transplantation model using Wistar rats as donors and Sprague-Dawley (SD) rats as recipients (**Supplementary Figures S1 A, B**), Allografts were harvested at postoperative days (POD) 0, 1, 3, and 7. CD45^+^ immune cells were isolated by fluorescence-activated cell sorting (FACS) and processed for single-cell RNA sequencing (10x Genomics) (**Figure 1A**). Concomitant histopathological analysis via H&E staining revealed progressive features of acute rejection over time, including escalating interstitial infiltration of mononuclear inflammatory cells, prominent tubulitis and intimal arteritis, along with increasing intracapillary inflammatory cell accumulation within glomerular tufts (**Figure 1B**), demonstrating the development of acute allograft rejection(**Supplementary Table 1**); Partial renal tubules exhibited hydropic degeneration of epithelial cells, disappearance of epithelial nuclei, and epithelial cell sloughing, culminating in necrosis of both glomeruli and tubules. Immunohistochemical staining revealed marked T-cell infiltration (*CD3*) and robust expression of *KIM-1* in the renal tubules (**Supplementary Figure S1C**), while biochemical analyses of serum creatinine and blood urea nitrogen (BUN) demonstrated significantly impaired renal function (**Supplementary Figure S1D**). Flow cytometric analysis corroborated these findings (**Figure 1C**), demonstrating a significant time-dependent increase in the proportion of infiltrating immune cells within renal allografts: 11.7% (Day 1), 20.9% (Day 3), 50.7% (Day 5), and 78.5% (Day 7).

### 3.2 scRNA-Seq reveals diverse immune cell types in renal allografts

**Figure 2:**
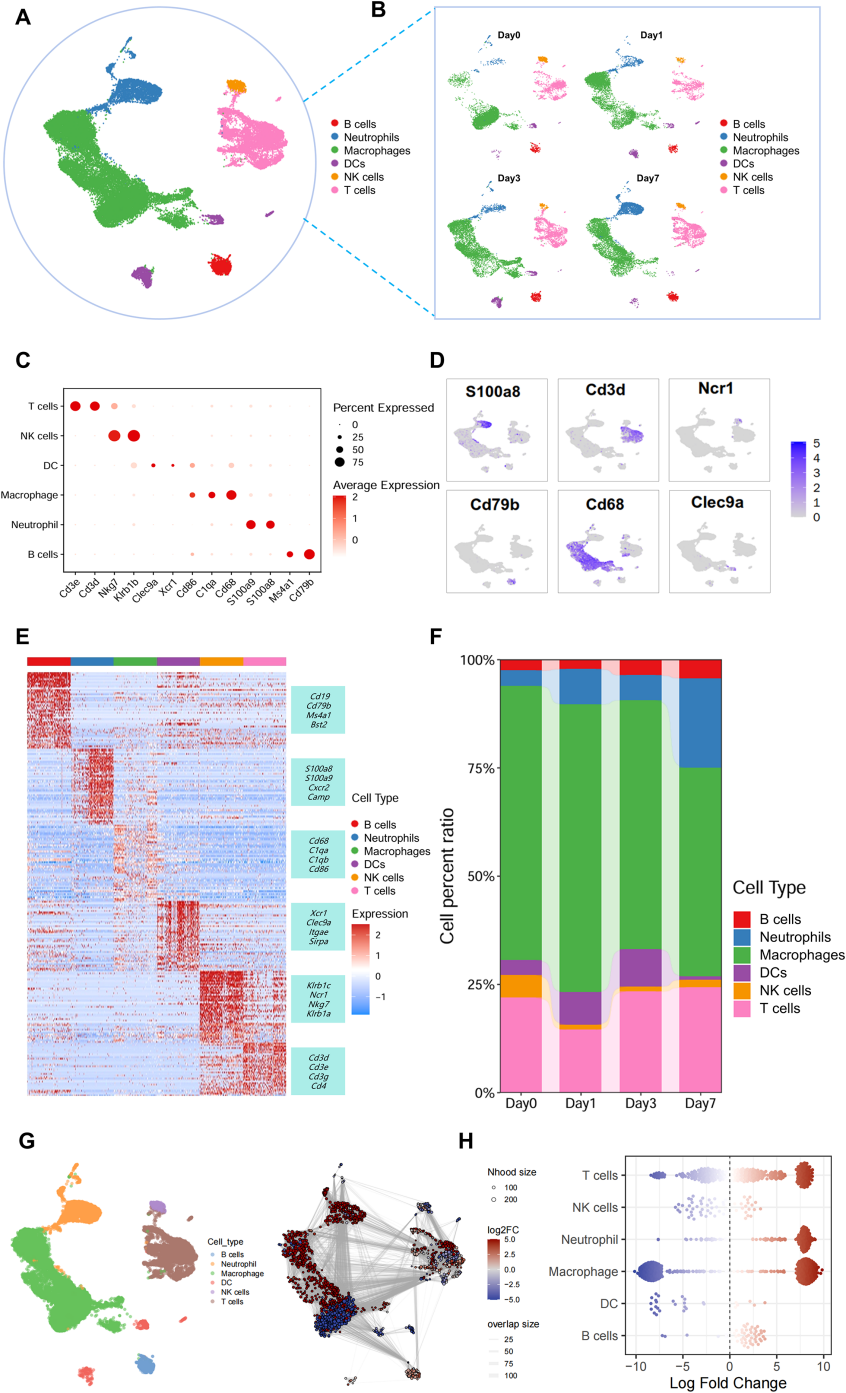
The early cellular dynamic changes in acute rejection of kidney transplantation. **(A)** UMAP visualization identifying six major immune cell types following unsupervised clustering. **(B)** Temporal stratification of immune cell types across time points. **(C)** Bubble plot depicting mean expression levels of canonical marker genes:B cells (*Ms4a1, Cd79b*), Neutrophils (*S100a8, S100a9*), Macrophages (*Cd68, Cd86, C1qa*), Dendritic cells(DCs) (*Clec9a, Xcr1*), Natural killer cells(NK cells) (*Klrb1b, Nkg7*) and T cells (*Cd3d, Cd3e*). **(D)** UMAP projection of canonical marker genes for the six major cell types. **(E)** Heatmap of the top 20 marker genes per cell population. **(F)** Bar plot illustrating proportional dynamics of each cell type across temporal samples. **(G)** Graph representation of neighborhoods identified by Milo. Nodes are neighborhoods, colored by their log fold change between Day7 and Day0 samples. Non-differential abundance neighborhoods (FDR 10%) are colored white, and sizes correspond to the number of cells in a neighborhood. Graph edges depict the number of cells shared between adjacent neighborhoods. The layout of nodes is determined by the position of the neighborhood index cell in the UMAP embedding of single cells. **(H)** Beeswarm plot showing the distribution of log fold change in abundance between conditions in neighborhoods from different cell type clusters. Differential abundance neighborhoods at FDR 10% are colored.

We obtained a total of 37,159 cells from all samples for single-cell RNA sequencing (scRNA-seq). Following rigorous quality control and robust batch effect removal/integration (**Supplementary Figures S2A, B**), cell populations were visualized in a Uniform Manifold Approximation and Projection (UMAP) plot using the Seurat R package. Unsupervised clustering revealed 19 distinct cell clusters (**Supplementary Figure S2C**). By annotating cell clusters through marker gene expression profiling and integration with published cell atlas datasets, we identified six major cell lineages across the four samples (**Figures 2A, B**), including B cells (*Ms4a1, Cd79b*), Neutrophils (*S100a8, S100a9*), Macrophages (*Cd68, Cd86, C1qa*), Dendritic cells(DCs) (*Clec9a, Xcr1*), Natural killer cells(NK cells) (*Klrb1b, Nkg7*) and T cells (*Cd3d, Cd3e*)(**Figures 2C–E**). Stream plot visualization revealed that macrophages constituted the predominant proportion among all immune cells. Innate immune cells, including NK cells and DCs, maintained relatively consistent proportions during the early phase (days 0, 1, and 3) but declined to minimal levels by day 7. In contrast, T cells and B cells—core components of adaptive immunity—progressively expanded following transplantation (**Figure 2F**). Differential abundance analysis using the MiloR package further revealed substantial increases in neutrophil, T cell, and B cell populations at day 7 post-transplantation during acute rejection (**Figures 2G, H**).

Collectively, our data clearly delineate the early-stage immune cell landscape in renal allograft acute rejection, revealing that macrophages may act as early “responders” in transplant immunity and play an important role in acute rejection.

### 3.3 T cell dynamics during early acute rejection

**Figure 3:**
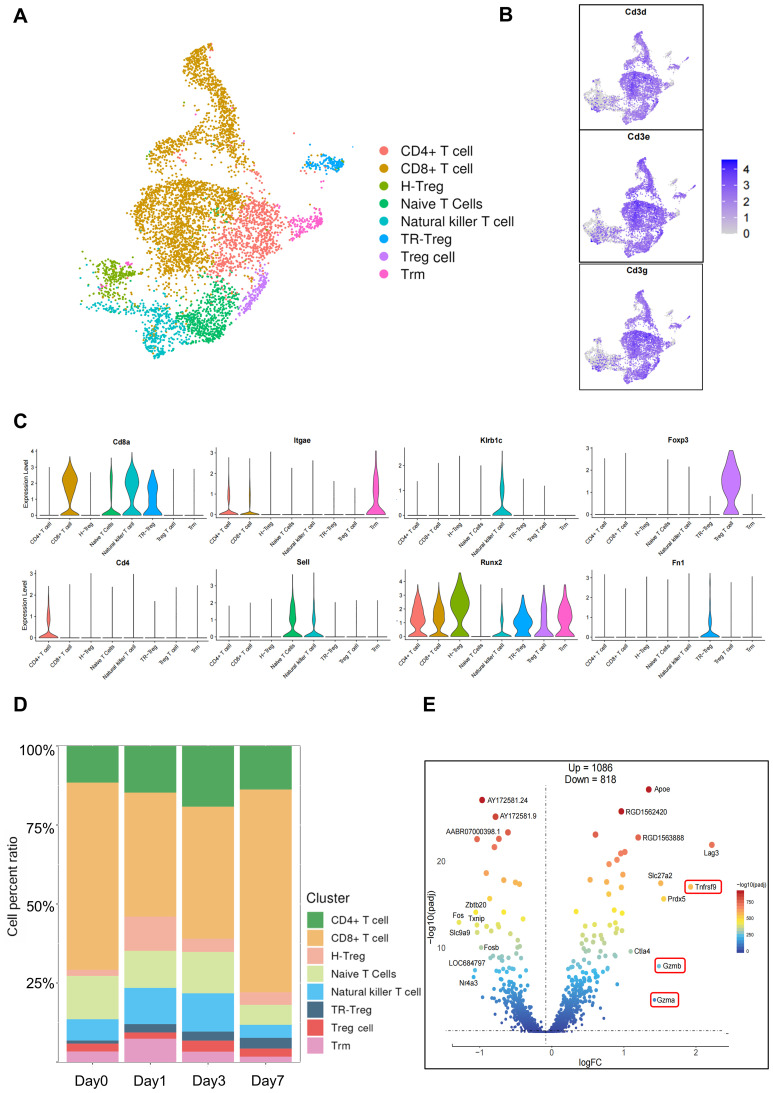
Sub-clustering of T cell. **(A)** UMAP plot of the pre-annotated macrophages. **(B)** UMAP projection of canonical marker genes for T cells. **(C)** Violin plot showing mean expression levels of cell type-specific canonical marker genes. **(D)** Bar plot illustrating proportional dynamics of T cell subsets across temporal samples. **(E)** Volcano plot displaying top 10 differentially expressed genes in T cells between post-transplant day 7 and non-transplanted controls.

Prior studies have established that T cells play a central role in mediating acute rejection, and their reactivity dictates both short- and long-term outcomes following solid organ transplantation (25). Therefore, we performed further subtyping of T cells and identified eight distinct T cell clusters (**Figures 3A, B**). By screening differentially expressed genes (DEGs) in each cluster and comparing them with previously reported T cell subsets and canonical marker genes in the literature, these eight clusters were defined as: CD4+ T cells (*Cd4, Cd40lg*); CD8+ T cells (*Cd8a, Gzma, Gzmb*); Natural killer T cells (*Klrd1, Klrb1c*); Treg cells (*Foxp3, Ctla4, Lag3*); Tissue-resident memory T cells(Trm);(*Cd69, Itgae [CD103]*); and Naive T cells (*Sell [CD62L]*). Cluster 3 was defined as “Homeostatic Regulatory T Cells” (H-Treg) due to its co-expression of genes associated with: negative regulation of signaling pathways-*Ptpn22* (a lymphoid tyrosine phosphatase that suppresses TCR signaling) and *Spred2* (an inhibitor of the Ras/MAPK pathway); And metabolic adaptation-*Foxo1* (regulating Treg metabolism and survival while inhibiting the PI3K-Akt pathway) and *Inpp4*b (a phosphoinositide phosphatase that dampens PI3K signaling); Cluster 6 was designated as “Tissue-Remodeling Regulatory T Cells” (TR-Treg) based on its co-expression of genes implicated in: inflammation modulation -*Il1b* (a pro-inflammatory cytokine), *Tgfbi* (mediating TGF-β signaling), and *Cd274* (PD-L1); And tissue remodeling-*Vcan* (versican) and *Fn1* (fibronectin) (**Figures 3C**, **S3A**).

Following the definition of T cell subsets, we constructed a river plot depicting the proportional abundance of each subset across time points (**Figures 3D**, **S3B**). This analysis revealed that CD4+ T cells characterized by an initial increase followed by a decline. In contrast, CD8+ T cells showed a marked expansion at Day 7, becoming the predominant T cell subtype. Furthermore, comparative analysis of differentially expressed genes in T cells at post-transplant day 7 versus day 0 via volcano plot revealed significant upregulation of cytotoxicity-associated genes, including granzyme A (*Gzma*), granzyme B (*Gzmb*), and *Tnfrsf9* (a co-stimulatory receptor; *CD137*) (**Figure 3E**). These findings collectively indicate that with the occurrence of acute rejection, effector T cells begin to activate and release cytokines as well as cytotoxic mediators, further leading to graft damage.

### 3.4 Heterogeneity in macrophage phenotypes and functions during acute rejection

**Figure 4:**
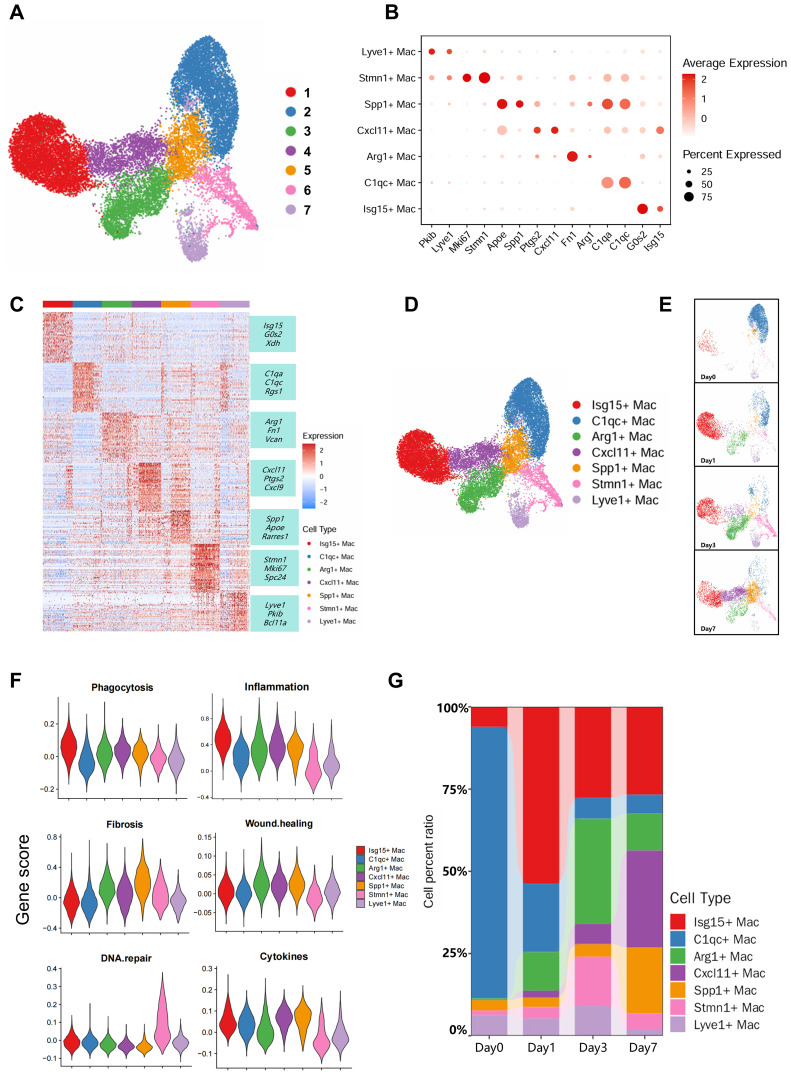
Sub-clustering of macrophage. **(A)** UMAP plot of the pre-annotated macrophages. **(B)** Bubble plot displaying expression of canonical marker genes associated with cell types. **(C)** Heatmap of top 20 marker genes in each sub-clusters. **(D)** UMAP plot of the post-annotated macrophages. **(E)** Stratification of macrophage subtypes by time point. **(F)** Comparative scoring of core functions across macrophage subsets (all time points). **(G)** Bar plot depicting proportional dynamics of macrophage subtypes across temporal samples.

Given the predominance of macrophages within the immune cell compartment, we performed macrophage subclustering to comprehensively investigate their heterogeneity and delineate the immune landscape during acute rejection, ultimately partitioning them into seven distinct subsets (**Figure 4A**). Each subset exhibited distinct transcriptomic signatures. Through differential gene enrichment analysis of macrophage subsets (**Figure 4C**), we generated bubble plots displaying subset-specific marker genes and visualized their expression patterns projected onto UMAP embeddings, successfully annotating these seven macrophage populations (**Figures 4B**, **S4A**). Additionally, functional profiling of each subset was performed using gene signature scoring (**Supplementary Table 1** (10)).

We defined cluster 1 as Isg15+ Mac because it expresses interferon-related genes (*Isg15*) and pro-inflammatory genes (*Nfkbiz, Tnfrsf1b, Xdh*), while simultaneously exhibiting the highest functional scores for phagocytosis and pro-inflammatory activity (26)(**Figure 4F**); Cluster 2 was defined as C1qc+ Mac (27), characterized by its expression of complement component genes (*C1qa, C1qc*); Cluster 3 was defined as Arg1+ Mac due to its expression of classical M2-type genes (*Arg1*) and tissue-repair genes (*Vcan*, *Fn1*, *Vegfa*) *(*28), while concurrently exhibiting the highest functional score for tissue repair promotion (**Figure 4F**); Cluster 4 was defined as Cxcl11+ Mac based on expression of the chemokines *Cxcl11* and *Cxcl9* (29); Cluster 5 was designated Spp1+ Mac due to its specific expression of *Spp1* (30), Scoring results indicate that this subset may promote tissue fibrosis;Cluster 6 characterized by high proliferative activity and elevated expression of proliferation-associated genes (*Stmn1*, *Mki67*, *Spc24*), was designated Stmn1+ Mac (26). Cluster 7 defined by *Lyve1* expression, was termed Lyve1+ Mac (31) (**Figure 4D**).

Finally, to provide an intuitive visualization of the dynamic changes in macrophage subsets following acute rejection, we generated time-stratified UMAP projections (**Figure 4E**) and stacked bar plots quantifying the proportional abundance of each subset across four post-transplantation time points (**Figure 4G**). We observed that C1qc+ Mac constituted a major proportion of macrophages in non-transplanted kidneys. Their abundance progressively decreased following acute rejection, suggesting a homeostatic role. Conversely, four subsets—Isg15+ Mac, Arg1+ Mac, Cxcl11+ Mac, and Spp1+ Mac—exhibited differential expansion during rejection. Notably, Isg15+ Mac demonstrated the most pronounced increase, with proportions surging within the first post-transplant day and sustaining elevated levels thereafter (**Figure 4G**). This indicates that the shift toward a pro-inflammatory macrophage phenotype occurs most rapidly following transplantation, manifesting within 24 hours.

### 3.5 Isg15+ Mac exhibited a distinct differentiation trajectory.

**Figure 5:**
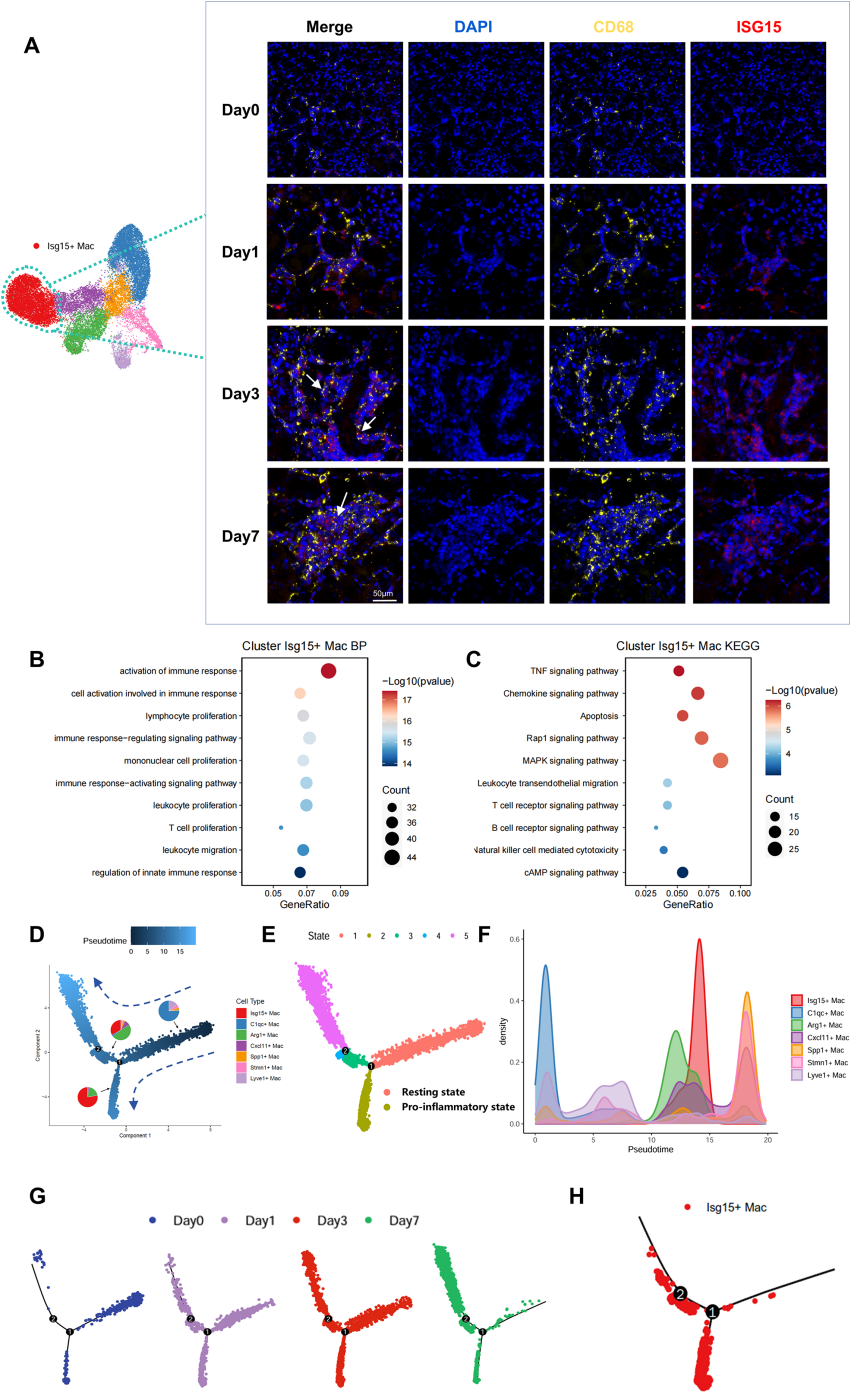
Macrophage trajectory dynamics during acute rejection. **(A)** Immunofluorescence of renal allografts at post-transplant days 0, 1, 3, and 7: Isg15+ Mac (red), CD68+ macrophages (yellow), DAPI (blue). Scale bars=50µm. **(B)** GO enrichment analysis of Isg15+ Mac. **(C)** KEGG pathway analysis of Isg15+ Mac. **(D)** Pseudotemporal trajectory plot with pie charts indicating subset proportions. Timeline scaled at top. **(E)** Trajectory map colored by cellular states, revealing activation paths. **(F)** Density plot of macrophage distribution along pseudotime. **(G)** Time-resolved stratification of pseudotemporal trajectory. **(H)** Isg15+ Mac cluster-specific trajectory mapping.

To validate the substantial presence of Isg15+ Mac post-transplantation, multiplex immunofluorescence staining for *Isg15* and *CD68* was performed. This analysis revealed significantly increased *Isg15* expression in renal allografts, with co-localization observed between *Isg15* and *CD68*, suggesting its expression on macrophage surfaces (**Figure 5A**). Isg15+ Mac also demonstrated high expression of genes critically involved in pro-inflammatory responses(*Il17ra* (32)、*Tnfrsf1* (33)、*Map4k1* (34)、*Itga4* (35)、*Irak3* (36)和*Xdh* (37))(**Supplementary Figure S4B**). We further performed GO enrichment analysis on macrophages(**Supplementary Figure S5A-B**), revealing that Isg15+Mac showed significant enrichment of genes involved in immune response activation—including activation of the immune response, lymphocyte proliferation, and T cell activation (**Figures 5B, C**). In summary, the Isg15+ Mac subset expands in renal allografts during acute rejection and demonstrates high expression of pro-inflammatory genes, indicating its critical involvement in acute rejection pathogenesis.

Next, trajectory analysis of macrophages was performed using Monocle 2, integrating functional annotations and differentially expressed genes across all subsets. This analysis designated State 1 as the initial state, while identifying C1qc+ Mac and Lyve1+ Mac as two macrophage subsets in a resting state (**Figures 5D–F**). This finding aligns with our observation that C1qc+ Mac predominates among macrophage subsets in Day 0 kidneys, reinforcing their role as a baseline population. Pseudotime analysis across temporal samples confirmed State 1 as the initial state, with cells progressively differentiating toward distinct trajectory endpoints (**Figure 5G**). Further stratification of the pseudotemporal trajectory by macrophage subsets revealed preferential localization of Isg15+ Mac within the State 2 branch (**Figure 5H**).

**Figure 6:**
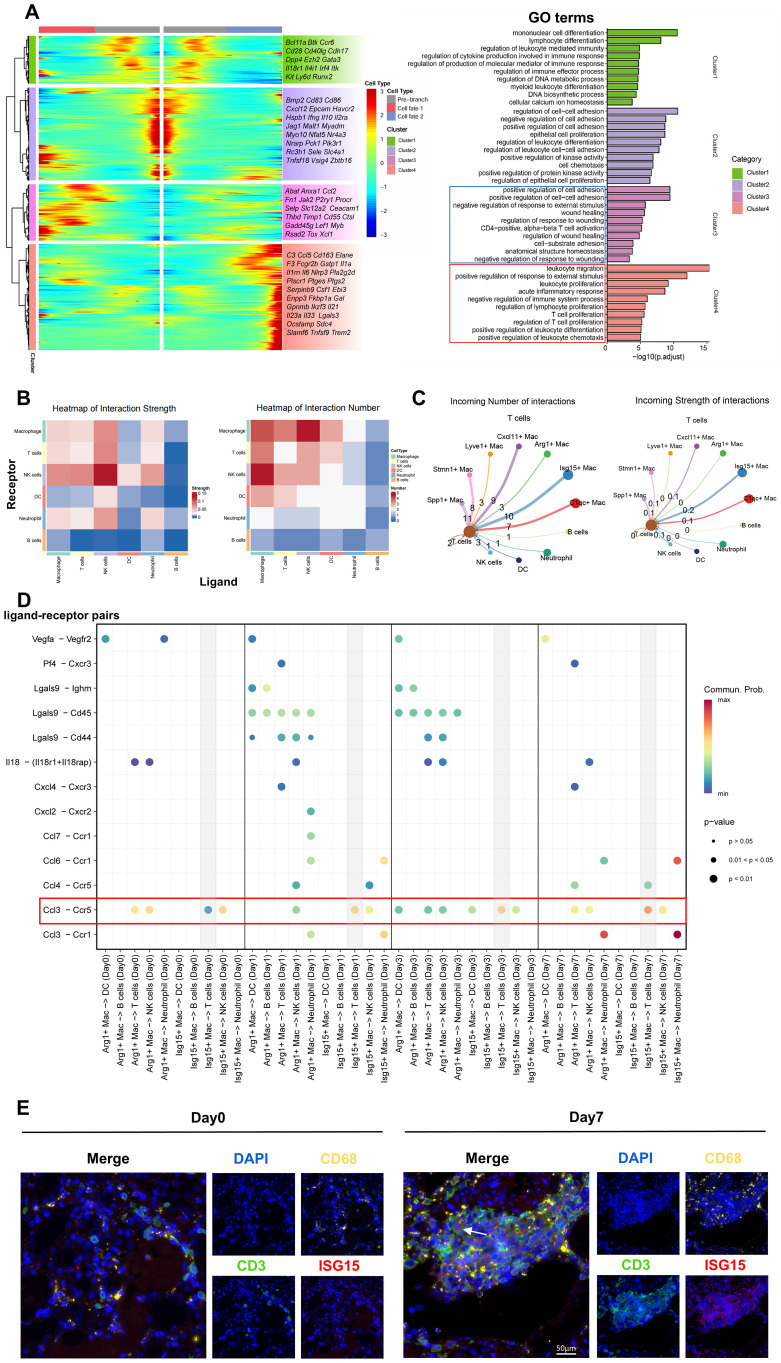
Ligand-receptor interactions in macrophage-centric cellular crosstalk. **(A)** Gene expression dynamics along pseudotime with parallel GO pathway enrichment analysis. **(B)** Heatmap of interaction quantity and intensity among major immune cell types. **(C)** Cell-cell communication network between macrophage subsets and T cells. Peripheral circle size denotes cellular abundance (larger = higher abundance); outbound arrows indicate ligand-expressing cells, inbound arrows denote receptor-expressing cells. Edge thickness corresponds to interaction weight. **(D)** Dot plot of selected ligand-receptor pairs between macrophage subsets (Isg15+ Mac, Arg1+Mac) and immune cells (dendritic cells, B cells, T cells, NK cells, neutrophils) across time points. **(E)** Multiplex immunofluorescence co-staining of T cells (CD3+) and Isg15+ macrophages (CD68+; Isg15+) in renal allografts. Scale bars=50µm.

Finally, differential gene expression analysis along the macrophage differentiation trajectory, coupled with GO functional enrichment, delineated the functional shifts during this process. We revealed that early in acute rejection, macrophages undergo a binary fate divergence: a pro-inflammatory trajectory and a pro-repair trajectory (**Figure 6A**). Specifically, Arg1+ Mac—representing the pro-repair branch—dominates post-injury tissue restoration, while Isg15+ Mac drives inflammation and rejection through the pro-inflammatory trajectory.

### 3.6 Cell-cell communication analysis identified the Ccl3-Ccr5 axis as a potential therapeutic target for enhancing renal allograft survival.

Using CellChat, we analyzed intercellular communication between macrophages and other immune cells. The intercellular communication heatmap revealed that macrophages primarily interact with T cells and NK cells (**Figures 6B**, **S6A**), which aligns with established literature reporting that macrophages infiltrate early post-transplantation and establish a pro-inflammatory microenvironment to activate T cells. Meantime, we validated the Ccl3-Ccr5 ligand-receptor pair as a critical signaling hub in macrophage-T cell communication (**Supplementary Figure S6B**). Next, we specifically investigated intercellular communication events with macrophage subsets as signal senders and T cells as receivers. This analysis revealed that Isg15+ Mac exhibited more prominent interactions with T cells—both in quantity and intensity—compared to other macrophage subsets (**Figures 6C**, **S7A–C**). Furthermore, the Ccl3-Ccr5 signaling axis played a dominant role in Isg15+Mac-T cell crosstalk, with signal strength progressively strengthening over time (**Figures 6D**, **S7D**). Co-localization of Isg15+ Mac and T cells in post-transplant renal allografts further supports their potential interactions (**Figure 6E**). Consequently, Isg15+Mac cells may act as early orchestrators of the immune response, influencing T-cell activation and recruitment following kidney transplantation through Ccl3–Ccr5 ligand–receptor interactions, and, concomitantly, releasing chemokines to recruit circulating peripheral T cells that infiltrate the graft and amplify the rejection response. Furthermore, by analyzing human renal biopsy specimens with rejection and acute kidney injury (AKI) (38), we also identified the abundant presence of Isg15+Mac, as well as the communication between Isg15+Mac and CD8+ T cells via the CCL3-CCR5 ligand-receptor pair (**Supplementary Figures S8A–D**).

**Figure 7:**
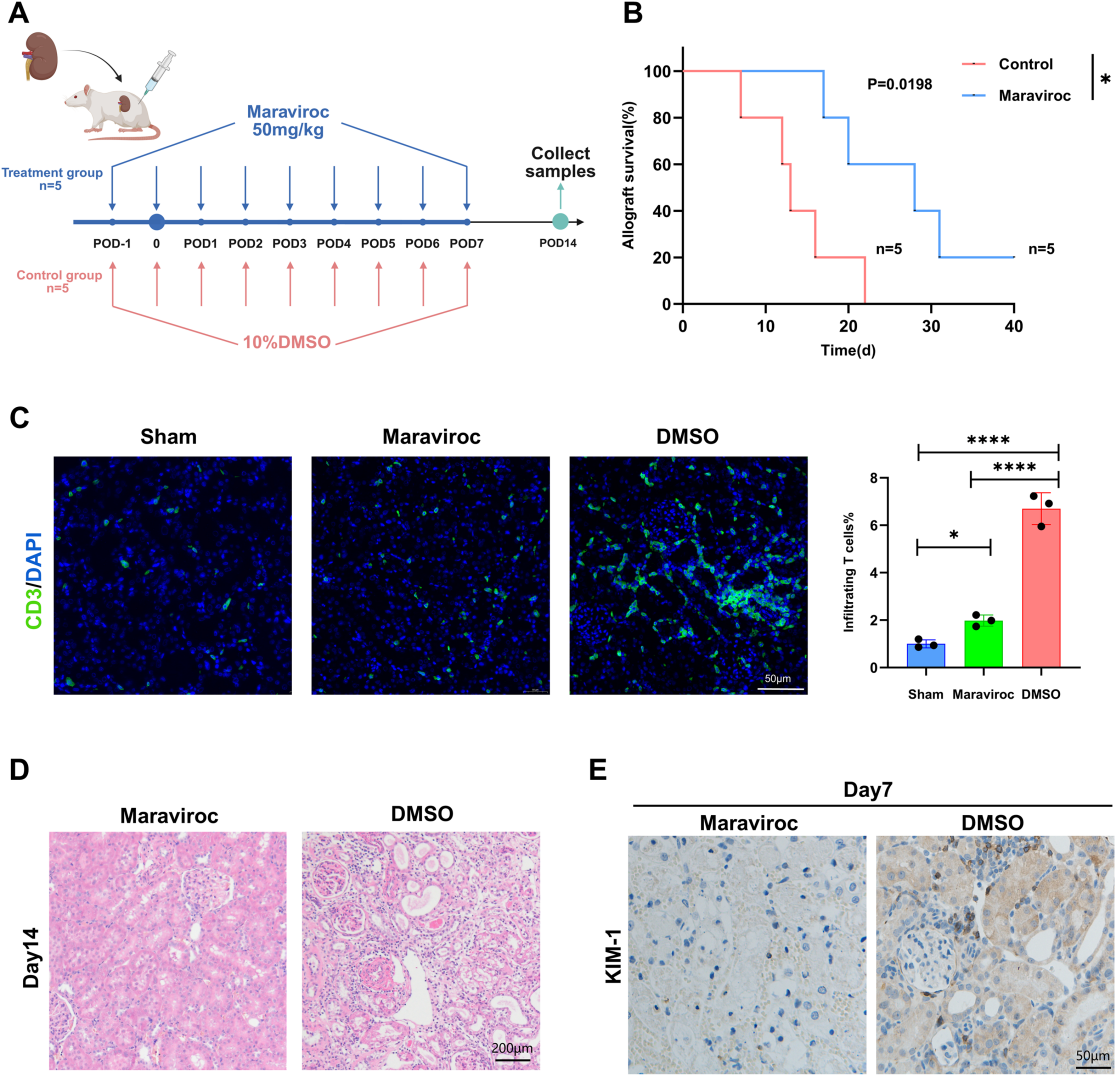
Maraviroc therapeutic intervention study. **(A)** Schematic of Ccr5 blockade protocol: Maraviroc administered 1 day preoperatively and daily post-transplantation (red arrows). **(B)** Kaplan-Meier survival curves of allografts with/without Maraviroc treatment (n=5 per group; endpoint = cardiacarrest; (*p<0.05), Gehan Breslow Wilcoxon test). **(C)** Left: Representative CD3+ T cells immunofluorescence in grafts (POD = 14) Scale bars=50μm; Right: Semiquantitative analysis of T cell-infiltrated areas (*p<0.05) (****p<0.0001). **(D)** Histological manifestations after Maraviroc treatment (POD = 14), Scale bars=200µm. **(E)** Immunohistochemical staining *KIM-1* after Maraviroc treatment (Day7), Scale bars=50µm.

As Ccr5 serves as the receptor for chemokines Ccl3, Ccl4, and Ccl5 and is highly expressed on T cells, we further validated the role of the Ccl3-Ccr5 axis in early renal allograft rejection. Rat kidney transplant recipients were randomized into two groups: the treatment group received Maraviroc (an FDA-approved Ccr5 antagonist) administered 1 day preoperatively and during the first postoperative week (**Figure 7A**), while the control group received an equivalent volume of DMSO. Maraviroc treatment significantly improved allograft survival in the treatment cohort (**Figure 7B**). Immunofluorescence confirmed reduced T cell infiltration in allografts (**Figures 7C**, **S9A**), Histological and immunohistochemical analyses of renal allografts demonstrated that renal injury was alleviated in the experimental group(**Figure 7D, E**), and renal function parameters showed partial recovery compared with the control group (**Supplementary Figure S9B**).

Collectively, we propose that Isg15+Mac may serve as critical contributors to acute rejection by activating T cells via the Ccl3–Ccr5 axis, thereby amplifying the process. Our results further indicate that targeting the Ccl3-Ccr5 axis represents a promising therapeutic strategy for preventing acute allograft rejection.

## 4 Discussion

With over 850 million people worldwide affected by kidney disease, end-stage renal disease (ESRD) constitutes a critical global public health challenge (39). While kidney transplantation offers the optimal therapeutic approach for ESRD, acute rejection remains a significant cause of early allograft loss (1, 3). Thus we leveraged single-cell RNA sequencing to map the dynamic immune landscape during the early phase (postoperative days 0, 1, 3, and 7) of acute rejection in a renal transplant model. scRNA-seq delineates the dynamics of immune cells and intercellular communication networks, not only decoding the covert immunological programs that initiate acute rejection but also revealing insights for early diagnosis and intervention. While T cells are established as central orchestrators of adaptive immunity-mediated allograft rejection (40)-a process inherently dependent on innate immune support (41)-the predominance of macrophages among innate immune cells in our scRNA-seq data prompted us to investigate whether this population could yield pathogenic insights into acute rejection. Thus, after identifying six major immune cell populations—T cells, B cells, NK cells, neutrophils, dendritic cells (DCs), and macrophages—we further refined our analysis to characterize T cell and macrophage subsets.

ScRNA-seq analysis revealed multiple T cell subsets within the allograft microenvironment. We identified eight distinct T cell populations: CD4+ T cells, CD8+ T cells, Natural killer T (NKT) cells, regulatory T (Treg) cells, tissue-resident memory T (Trm) cells, and naive T cells; Furthermore, differential gene expression analysis enabled annotation of two novel regulatory T cell subsets: Homeostatic Regulatory T Cells (H-Treg) and Tissue-Remodeling Regulatory T Cells (TR-Treg). Among these subsets, CD8+ T cells remained the dominant population, progressively expanding during rejection. Comparative transcriptomic analysis of pre- and post-transplant T cells further revealed significant upregulation of cytotoxic mediators—*Gzma, Gzmb*, and *Tnfrsf9*—corroborating the gradual activation of adaptive immunity.

In renal transplantation, macrophages have historically been categorized into M1/M2 phenotypes. However, this binary classification represents an oversimplification of their functional plasticity during rejection (14, 42, 43). With the application of scRNA-seq in transplantation, increasing novel macrophage phenotypes are being discovered, such as classifying macrophages into donor-derived and recipient-derived. Depletion of donor MCR2+ macrophages can prolong allograft survival (44); Ning et al. observed a higher proportion of FCN1+ macrophages in ABMR patients compared to non-rejection controls, suggesting their potential role in ABMR (45); Zhu et al. identified a novel Myoz2+ macrophage subset uniquely expressing cardiac contraction-related genes in murine renal allografts (46). Through subclustering analysis, we identified seven macrophage subsets and discovered that Isg15+ Mac likely represents a rejection-associated population. This subset exhibited high expression of pro-inflammatorygenes (*Il17ra*, *Tnfrsf1b*, *Map4k1*, *Itga4*, *Irak3*,

*Xdh*). Concurrently, Isg15+ Mac underwent substantial expansion as early as postoperative day 1, maintaining stably elevated proportions through days 3 and 7. Multiplex immunofluorescence corroborated these findings, Functional profiling further demonstrated its pivotal role in promoting immune activation, inflammatory tissue injury, and T cells recruitment. Previous studies have documented Isg15+ macrophages in pulmonary and cardiac tissues, where their accumulation correlates with impaired tissue repair and progressive organ dysfunction (26, 47); Concurrently, Zhang et al. reported sustained differential expression of Isg15 in circulating monocytes from renal allograft recipients experiencing rejection compared to healthy controls, demonstrating its potential as a diagnostic biomarker for transplant rejection (48).

Subsequent analysis of macrophage-T cell crosstalk revealed that Isg15+ Mac engages with T cells through the Ccl3-Ccr5 ligand-receptor pair. Immunofluorescence co-localization further corroborated potential interactions between these populations. Finally, pharmacological blockade of the Ccl3-Ccr5 ligand-receptor axis using Maraviroc—an FDA-approved Ccr5 antagonist—significantly improved renal allograft survival, attenuated T cells infiltration within grafts, and mitigated histological manifestations of acute rejection. We therefore propose the Isg15+ macrophage subset as “orchestrators” in the early phase of acute rejection following kidney transplantation, which, by establishing a proinflammatory microenvironment and efficiently presenting antigens as well as secreting chemokines such as Ccl3, amplify the magnitude and intensity of subsequent T-cell responses.

Ccr5 is a G protein-coupled receptor (GPCR) that functions as a co-receptor for HIV-1 entry. It binds multiple chemokines, including Ccl3, Ccl4, and Ccl5, to mediate inflammatory signaling (49). This receptor is predominantly expressed on T cells, where it mediates T cells adhesion and migration (50). Ccr5 deficiency has been shown to impair T cells memory responses and antigen responsiveness (51). Several studies have implicated the Ccr5 signaling axis in allograft rejection (52–54), identifying it as a diagnostic biomarker in renal transplant recipients experiencing rejection; He et al. reported recruitment of Ccr5+ T cells in human acute renal allograft rejection. In our study, blockade of the specific T-cell recruitment pathway driven by Ccl3 derived from Isg15+Mac inhibited T-cell infiltration and activation, thereby attenuating transplant rejection.

Maraviroc—the first FDA-approved Ccr5 antagonist for HIV treatment and an emerging agent in certain anticancer regimens—directly binds to Ccr5, preventing its internalization and inhibiting T cells chemotaxis and migration (21). While the therapeutic potential of Maraviroc in transplantation remains underexplored, in our study, we adopted a prophylactic regimen with Maraviroc (administered via intraperitoneal injection at 50mg/kg once daily, starting 1 day before surgery). This approach achieved saturation of Ccr5 receptors prior to Isg15+Mac-mediated T cells activation, successfully alleviating the occurrence of acute renal allograft rejection in rats and reducing T cells infiltration. Compared with broad-spectrum T-cell depletion therapy, targeting the Ccl3–Ccr5 axis may offer a more precise immunotherapeutic strategy. Rather than depleting all T cells, this approach specifically blocks the pathological T-cell recruitment and activation pathway driven by a distinct macrophage subset. Moreover, as an approved drug, maraviroc has a well-defined safety profile and holds rapid translational potential for “drug repurposing” (55).

Despite its novel findings, this study has several limitations: 1) Further mechanistic investigations are required to elucidate how Isg15+ Mac instigates inflammatory cascades and orchestrates complex intercellular interactions within the renal acute rejection microenvironment. 2) When blocking Ccr5 with Maraviroc, it is still necessary to clarify its effects on kidney tissue cells and other immune cells, to provide sufficient basis for clinical translation. 3) Our study focused on CD45^+^ immune cells to achieve high-resolution analysis of immune heterogeneity. While this approach enabled detailed characterization of immune subpopulations, it did not assess contributions from parenchymal cells. Future spatial transcriptomics studies will be essential to elucidate the complete cellular crosstalk within renal allografts. 4) Finally, this paper is based on single-cell analysis of a rat kidney transplant model. In the future, we will further verify our findings in human kidney transplant rejection specimens.

This study reveals a novel pro-inflammatory macrophage subset (Isg15+ Mac) that mounts an ultra-rapid response during the earliest phase of transplantation, establishes the Ccl3-Ccr5 axis as a pivotal therapeutic target for enhancing renal allograft survival, and demonstrates considerable clinical translational promise for Maraviroc. In summary, our study provides an in-depth, comprehensive atlas of the immune landscape in renal transplantation. By sampling at different time points, we can better understand the occurrence and development of acute rejection, help develop more precise treatment strategies in the future and further improve long-term graft survival rates.

## Data Availability

The datasets presented in this study can be found in online repositories. The names of the repository/repositories and accession number(s) can be found in the article/**Supplementary Material**.
