# Correction: Single-cell transcriptomics unravels the early immune landscape of renal allograft rejection and nominates Ccl3-Ccr5 as a therapeutic target

**DOI:** 10.3389/fimmu.2025.1740964

**Published:** 2025-11-21

**Authors:** Fanchao Wei, Zhaoxiang Wang, Ruochen Qi, Jingliang Zhang, Shichao Han, Changhong Shi, Tong Lu, Zhite Zhao, Zhengxuan Li, Lang Li, Weijun Qin, Shuaijun Ma, Lijun Yang

**Affiliations:** 1 Department of Urology, Xijing Hospital, Air Force Medical University, Xi’an, Shaanxi, China; 2 Division of Cancer Biology, Laboratory Animal Center, Air Force Medical University, Xi’an, Shaanxi, China; 3 Skills Training Center, Xijing Hospital, Air Force Medical University, Xi’an, Shaanxi, China

**Keywords:** kidney transplant, acute rejection, macrophages, ScRNA-seq, Cc3-Ccr5

**Supplementary material: Supplementary Datasheet 1**, the GEO Series accession number for the data source from the supplementary materials was omitted. The file has now been updated and published.

The original version of this article has been updated.

