# Supplementary material for: Single-cell transcriptomics unravels the early immune landscape of renal allograft rejection and nominates Ccl3-Ccr5 as a therapeutic target
Source: Front Immunol. 2025 Oct 22;16:1663251. doi: 10.3389/fimmu.2025.1663251 (PMC12586113; doi:10.3389/fimmu.2025.1663251)
Supplement: Supplementary file 1 [file DataSheet1.docx]

**Supplementary Material**

**Data availability statement**

**Supplementary Figure 1**

**Supplementary Figure 2**

**Supplementary Figure 3**

**Supplementary Figure 4**

**Supplementary Figure 5**

**Supplementary Figure 6**

**Supplementary Figure 7**

**Supplementary Figure 8**

**Supplementary Figure 9**

**Supplementary Table 1**

**Supplementary Table 2**

**Data availability statement:**

The single-cell sequencing datasets generated and analyzed during the current study are being deposited in the Gene Expression Omnibus (GEO) repository: <https://www.ncbi.nlm.nih.gov/geo/query/acc.cgi?acc=GSE303904>

The human kidney biopsy single-cell RNA sequencing data used in this study have been deposited in the NCBI database under the accession number GSE145927.

**Supplementary Figure 1:**

Sample preparation and data integration.

(A) Opening of the blood flow in the transplanted kidney.

(B) Ultrasound examination of the blood flow in the transplanted kidney.

(C) Immunohistochemical staining of *CD3* and *KIM-1* in renal tissue at day 7 post-transplantation Scale bars=50µm.

(D) Changes in serum creatinine and blood urea nitrogen (BUN) in rats at day 7 post-transplantation(*p<0.001).

**Supplementary Figure 2:**

(A) Before batch effect corrected, scatterplot illustrating the number of genes, unique molecular identifiers (UMIs) and percentage of mitochondrial genes in the four groups.

(B) After batch effect corrected, scatterplot illustrating the number of genes, unique molecular identifiers (UMIs) and percentage of mitochondrial genes in the four groups.

(C) The unsupervised clustering results identified a total of 19 cell subpopulations.

**Supplementary Figure 3:**

(A) Bubble plot displaying expression of canonical marker genes associated with T cell subtypes.

(B) Bar plot illustrating dynamic changes in the proportions of CD4+ and CD8+ T cell subsets across temporal samples.

**Supplementary Figure 4:**

(A) UMAP projection of macrophage subsets based on subset-specific marker gene expression.

(B) Expression of pro-inflammatory genes in Isg15+ macrophages.

**Supplementary Figure 5:**

(A) GO enrichment analysis of biological processes across macrophage subsets.

(B) KEGG pathway enrichment analysis in macrophage subsets.

**Supplementary Figure 6:**

(A) Cell-cell communication analysis. Chord diagram depicting interaction strength and quantity from macrophage senders to receiver immune cells.

(B) Dot plot visualizing ligand-receptor pairs mediating intercellular interactions across time points.

**Supplementary Figure 7:**

(A) Chord diagram of cell-cell communication analysis depicting interaction number versus weight.

(B) Chord plot visualizing signal quantity received by immune subsets.

(C) Chord plot displaying signal strength received by immune subsets. The size of peripheral colored circles represents cellular abundance (larger size indicates higher cellular abundance). Cells expressing ligands are denoted by outbound arrows; receptor-expressing cells feature inbound arrows.

(D) The relative contributions of various chemokine ligand-receptor pairs to global signaling pathways following renal transplantation.

**Supplementary Figure 8: scRNA-Seq data of Renal Allograft Patients.**

(A) Identification of the major cell components in transplanted kidney.

(B) UMAP Plots show the distribution of macrophages subtypes.

(C) Bar plot illustrating the proportions of macrophage subsets across five samples. (D) Dot plot of ligand-receptor pairs between Isg15+ Mac and immune cells T cells.

**Supplementary Figure 9:**

(A) Multiplex immunofluorescence co-staining of T cells (CD3+) and Isg15+ macrophages (CD68+; Isg15+) in renal allografts.Left: Maraviroc treatment group; Right: DMSO control group. Scale bars=50µm.

(B) Serum creatinine and blood urea nitrogen (BUN) levels in rats after treatment with Maraviroc. (*p<0.001).

**Supplementary Table 1:**

| **Post-transplant histopathological evaluation according to the Banff criteria** | | | | |
| --- | --- | --- | --- | --- |
| **Banff lesion score** | Day0 | Day1 | Day3 | Day7 |
| Interstitial inflammation | i0 | i1 | i2 | i2 |
| Tubulitis | t0 | t1 | t1 | t2 |
| Intimal arteritis | v0 | v1 | v1 | v1, v* |
| Glomerulitis | g0 | g1 | g1 | g3 |
| Peritubular capillaritis | ptc0 | ptc1 | ptc2 | ptc3 |

**Supplementary Table 2:**

| Genes used for gene scores analysis. | |
| --- | --- |
| **Scores** | **Genes** |
| Fibrosis | *Pdgfb,Tgfb1,Col3a1,Acta2,Mmp2,Pdgfa,Vim,Nkd2,Tnc,Gpnmb,Cd9,Tnfsf12,Tl2r,Tnf,Igf1,Timp2,Anxa5* |
| Inflammation | *Cd74,Tnfrsf12a,Cxcl1,Cxcl10,Cxcl16,IL1b,Cxcl2,Ccl3,Tyrobp,C3,Ccl5,Ccl7,Ccl8,Ccl12,Lcn2,S100a6,Cd40,Ifit1,Il6,Tnf,Ifng,Vcam1,Icam,Ccl2,Cxcl3,Ccr2,Cxcr2,Ccn2,Trem2,Il17ra,Nfkbiz,Cebpb,Tnfrsf1b,S100a4,Map4k1,Xdh,Irak3,Itga4,Itgb2,Gch1,Sat1,Ifit2,Ifit3,Nod2,Isg15,Irf1* |
| Phagocytosis | *Irf8,Lyst,Hck,Abr,Lepr,Sirpb1a,Elmo3,Met,Mertk,Rab11fip2,Tafa4,Itgb2,Itgb1,Itgam,Itgal,Abca7,Lrp1,Tlr4,Anxa1,Bltp1,Ldlr,Lep,Sh3bp1,Myo7a,Megf10,Gata2,Gas6,Tusc2,Axl,Mst1r,Cebpe,Anxa3,Elmo2,Elmo1,Mex3b,Elane,Cdc42se2,Slc11a1,Ncf2,P2ry6,Tyro3,Pld4,Pik3ca,Cnn2,Myd88,Anxa11,Eif2ak1,Tub,Vav1,Pip5k1c,Dnm2,Bcr,Cryba1,Tm9sf4,Ncf4,Dock1,Icam5,Jmjd6,Rap1gap,Tulp1,Slamf1,Unc13d,Prtn3,Fcgr2b,Nod2,Mesd,Adgrb1,Gulp1,Rab5a,Abl2,Abl1,Myo1g,Cd302,Pecam1,Cdc42se1,Ticam2,Coro1c,Coro1a,Pla2g5* |
| Wound healing | *Hps4,Arhgef19,Arhgap24,Cldn1,Fkbp10,Gp9,Ppara,Bloc1s4,Scnn1b,Scnn1g,Enpp4,Cav3,Evpl,Lyst,Stard13,Nrg1,Hbegf,Ptpn6,Serpind1,Gp1bb,Tubb1,Ndnf,Myh9,Acvrl1,Proz,Plek,Chmp4b,Ajuba,C9,Clec10a,Mertk,Vps4a,Nog,Pik3cb,Kdr,Itgb3,Itga2b,Sytl4,Ins2,Ins1,Il1a,Igf1,Tlr4,Lox,Dst,Procr,Ptprj,Dtnbp1,Fermt3,Rap2b,Cx3cl1,Fer1l5,F10,Papss2,Gata4,Gata2,Gata1* |
| DNA repair | *Irf8,Bub3,Cenpb,Cenpa,Mki67,Ndc80,Cenpf,Pmf1,Incenp,Nuf2,Smc6,Csnk1a1,Nde1,Cdt1,Hells,Top2a,Spdl1,Pkhd1,Cenpq,Ska3,Ercc6l,Cbx5,Nek2,Dync1li1,Kat7,Spag5,Kif2b,Cenps,Sycp3,Nup43,Trp53bp1,Cenpv,Ctcf,Suv39h1,Ckap5,Clasp1,Rassf2,Spc25,Cenpo,Cenpp,Cenpw,Sycp1,Smc3,Zfp330,Clasp2,Knl1,Septin6,Dctn5,Cenpl,Zw10,Ppp2r5c,Cenph,Hnrnpu,Mis18a,Cenpm,Dapk3,Birc5,Mis18bp1,Ska1,Kansl1,Zfp276,Nudcd2,Ska2,Dsn1,Spout1,Cdca8,Zfp207,Nup85,Hsf1,Ss18l1,Cenpc1,Aurkb,Smc5,Cenpx,Ahctf1,Dync1i1,Anapc16,Champ1,Cenpt,Phf6,H3f3a,Ppp2r5a,Kat5* |
| Cytokines | *Tnf,Tnfrsfla,Tnfrsf1b,Il1b,Il6,Il10,Icam1,Vcam1,Ccl5,Cxcl2,Ccl2,Il1a,Il18,Ccl12,Ccl7,Ccl8,Cx3cl1,Il34,Pdgfa,Pdgfb,Pdgfd,Tgfb2,Ifng,Il1rn,Il2,Il4,Il5,Il7,Il9,Il12b,Il13,Il15,Cxcl9,Cxcl10,Ccl3,Ccl4,Ltb,Il16,Hmgb1,Grn,Tnfsf10,Cklf,Timp1,Aimp1,Cmtm3,Tnfsf12,Cmtm7,Nampt,Tgfb1,Tnfsf14,Tnfsf13b,Ccl1,Il15,Il16,Il17a,Il17b,Il17c,Il17d,Il17f,Il18,Il19,Ccl11,Il2,Il20,Il21,Il22,Il23a,Il24,Il25,Ccl12,Il27,Ebi3,Ifnl2,Ifnl3,Il3,Il31,Il33,Il34,Il4,Il5,Il6,Il7,Il9* |
