# Supplementary figures and images for: Single-cell transcriptomics unravels the early immune landscape of renal allograft rejection and nominates Ccl3-Ccr5 as a therapeutic target
Source: Front Immunol. 2025 Oct 22;16:1663251. doi: 10.3389/fimmu.2025.1663251 (PMC12586113; doi:10.3389/fimmu.2025.1663251)

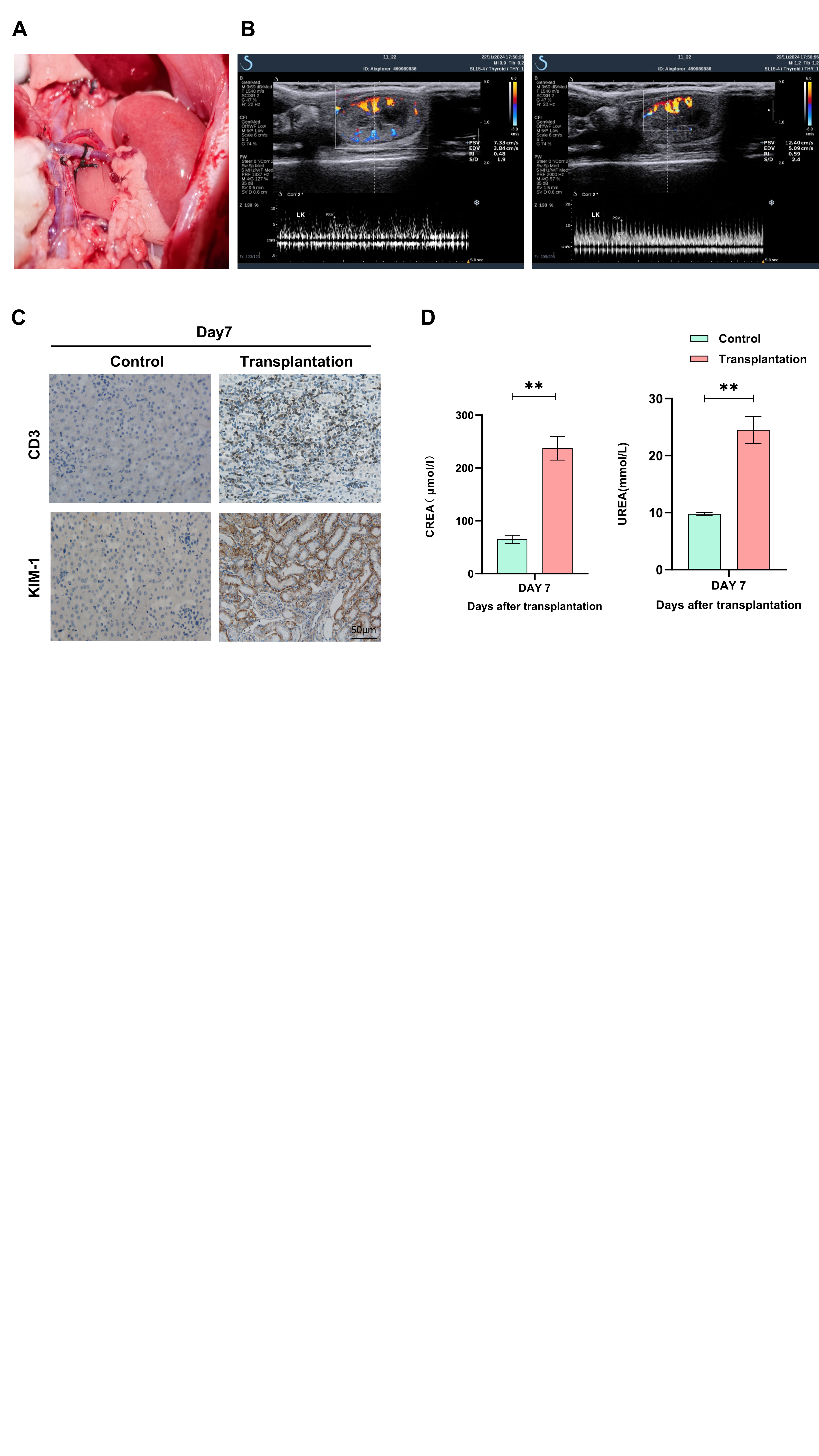

Supplement: Supplementary file 4 [file Image1.tif]

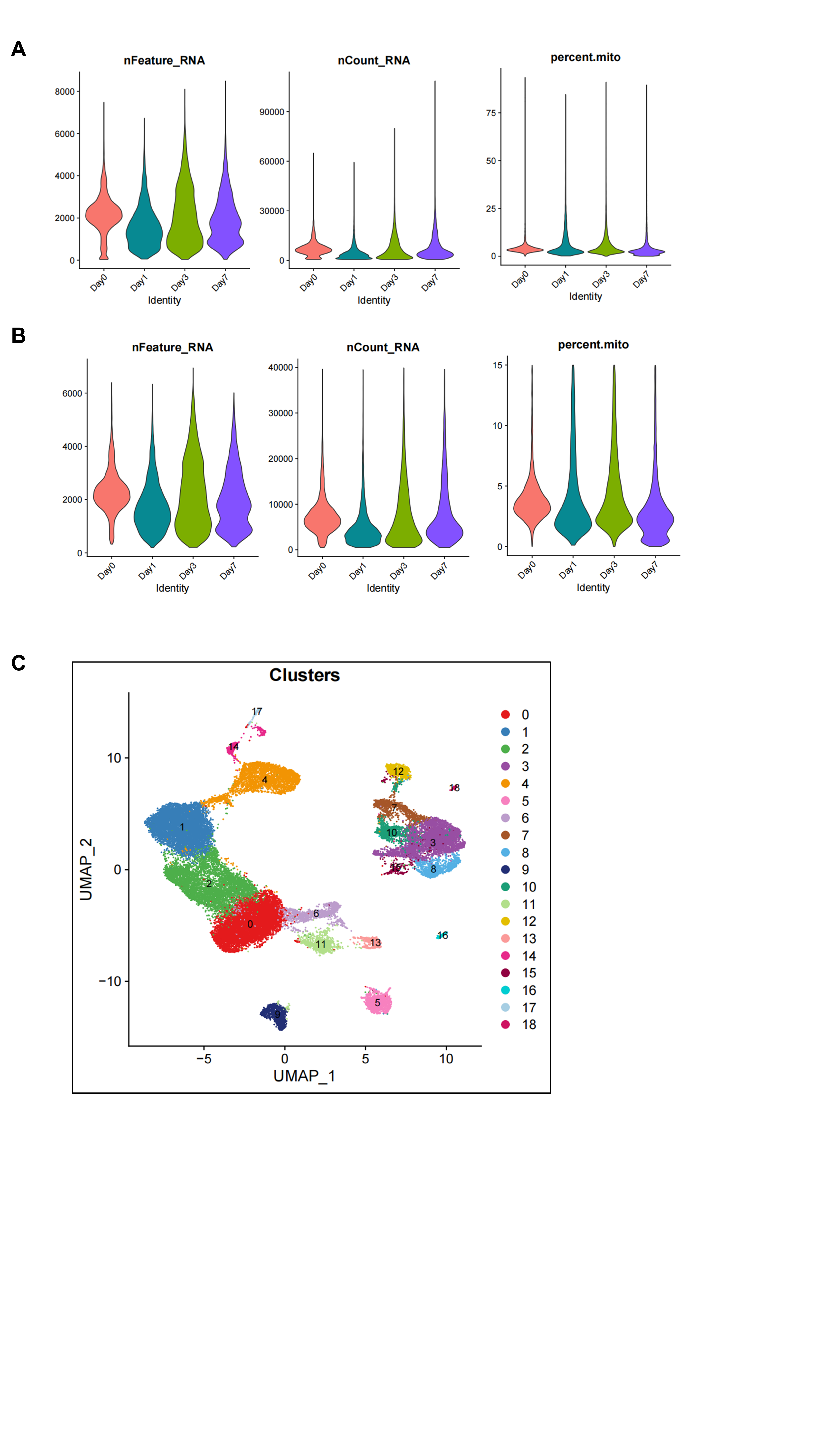

Supplement: Supplementary file 5 [file Image2.tif]

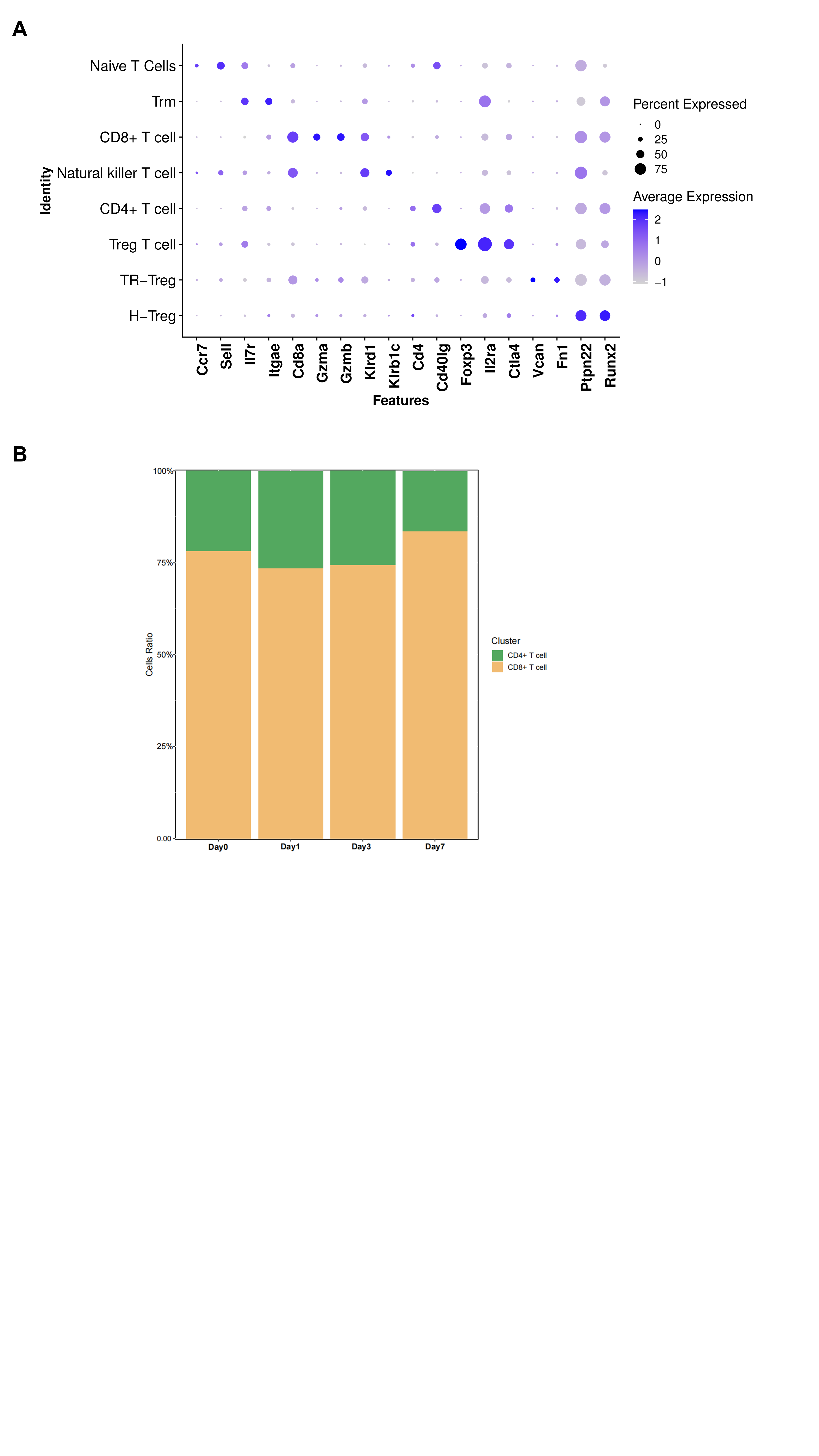

Supplement: Supplementary file 6 [file Image3.tif]

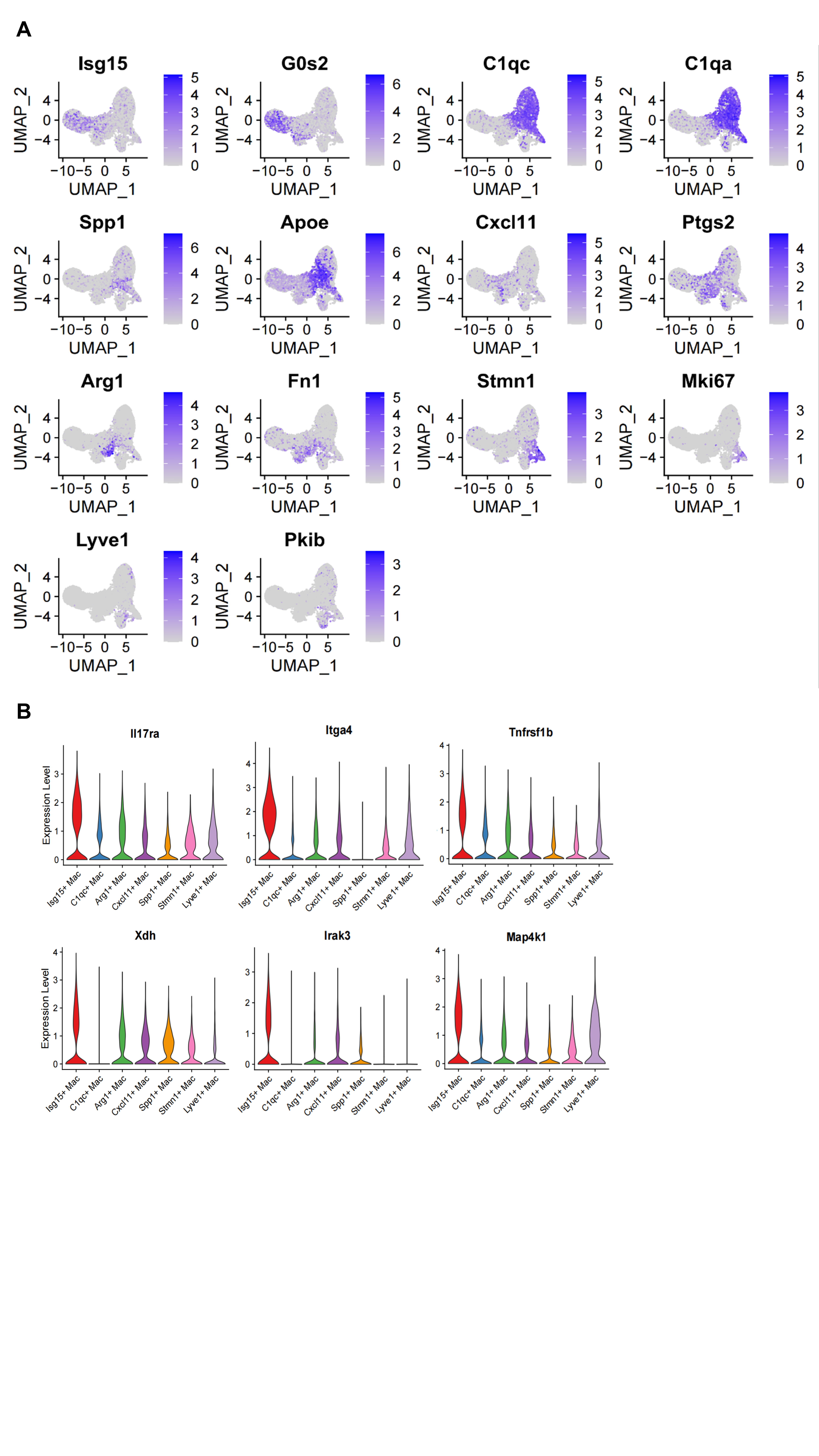

Supplement: Supplementary file 7 [file Image4.tif]

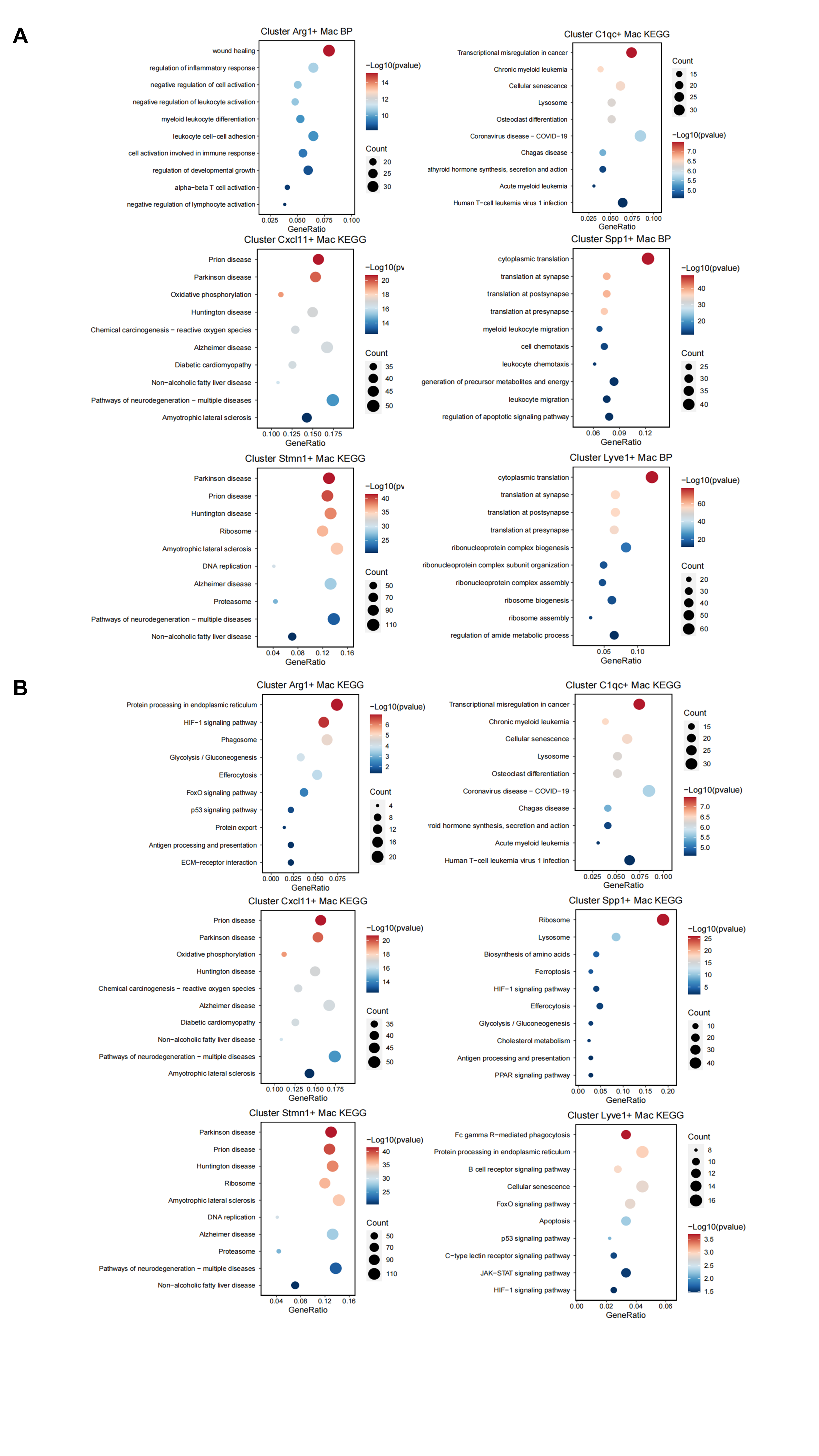

Supplement: Supplementary file 8 [file Image5.tif]

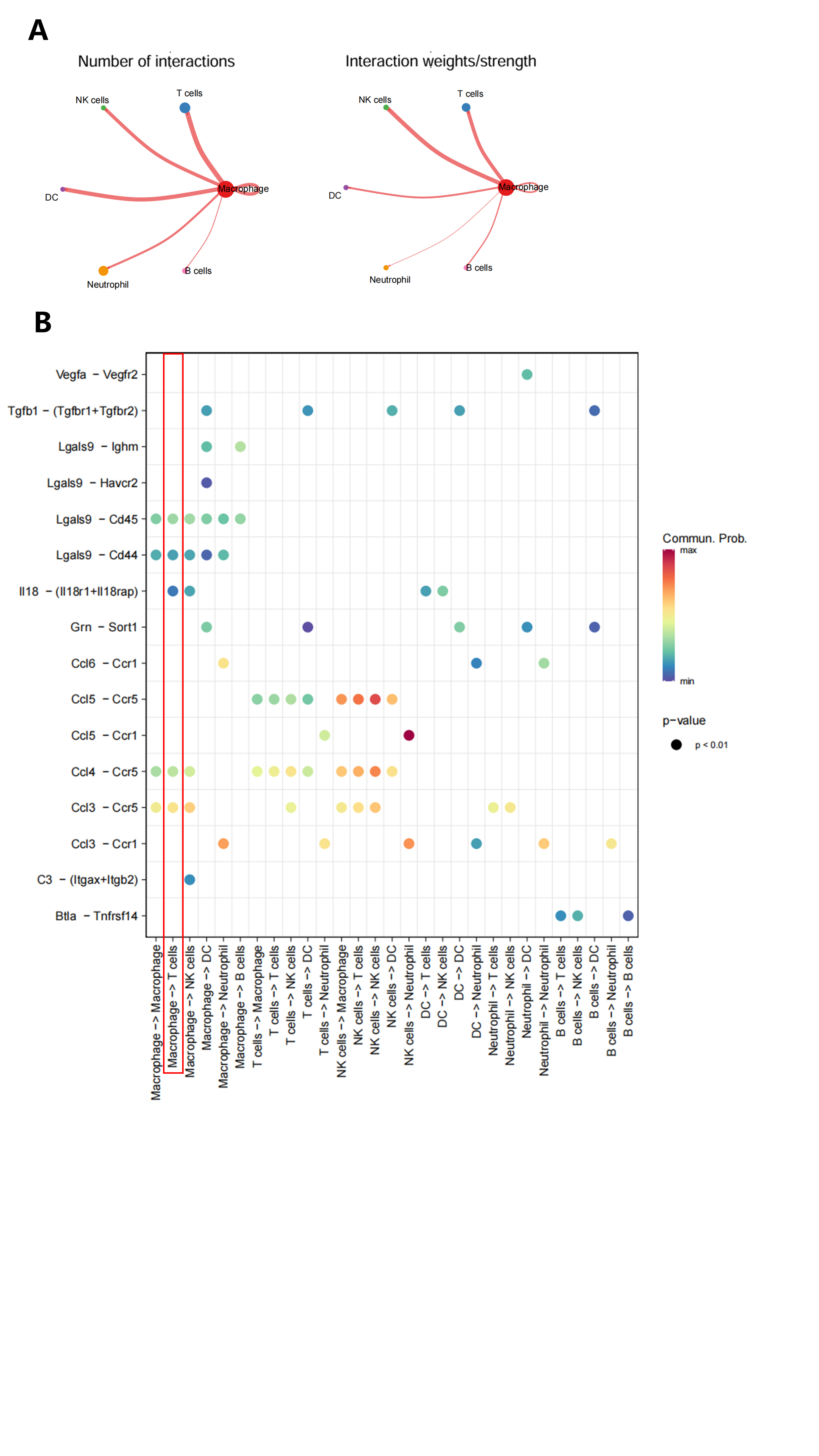

Supplement: Supplementary file 9 [file Image6.tif]

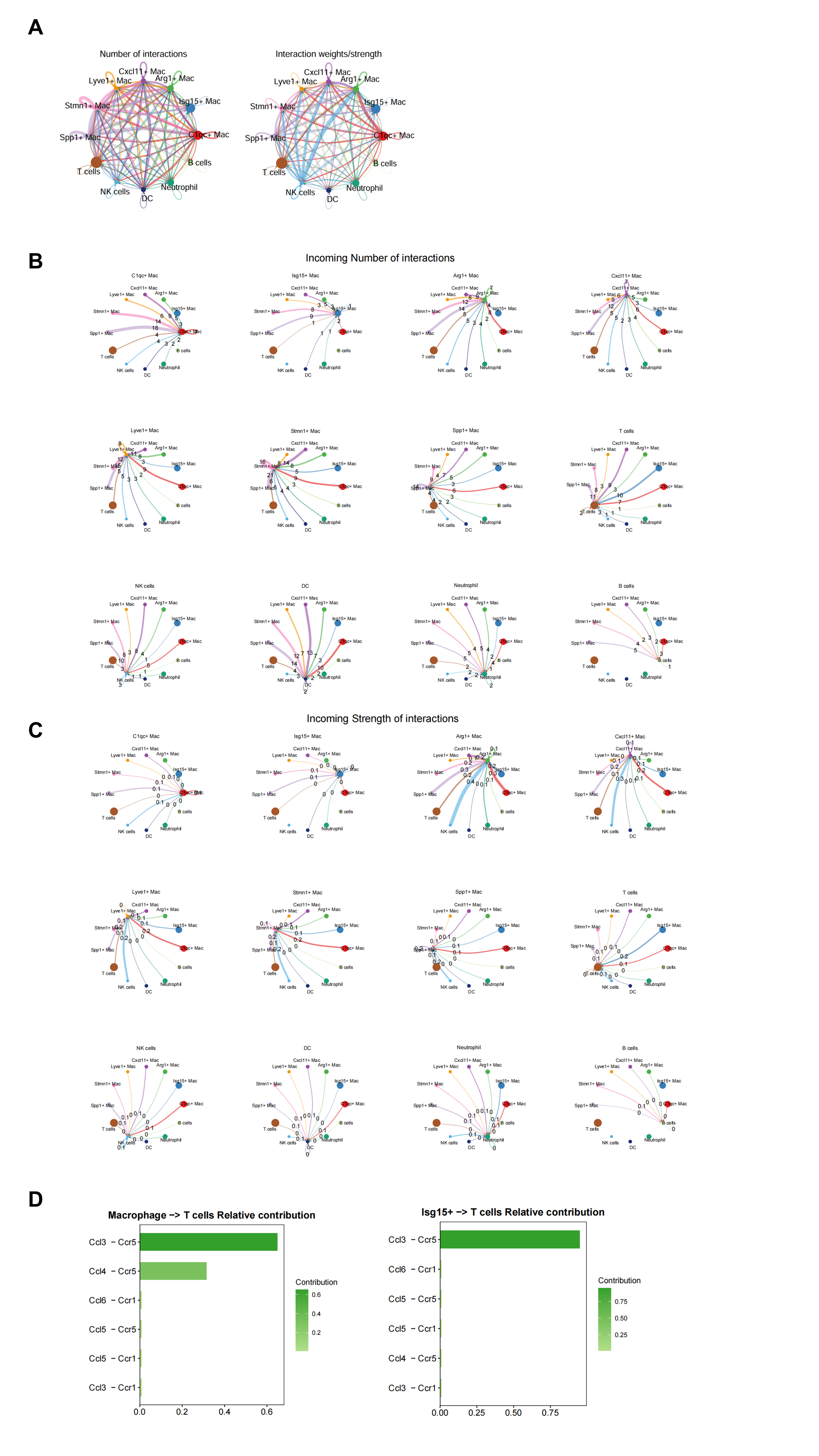

Supplement: Supplementary file 10 [file Image7.tif]

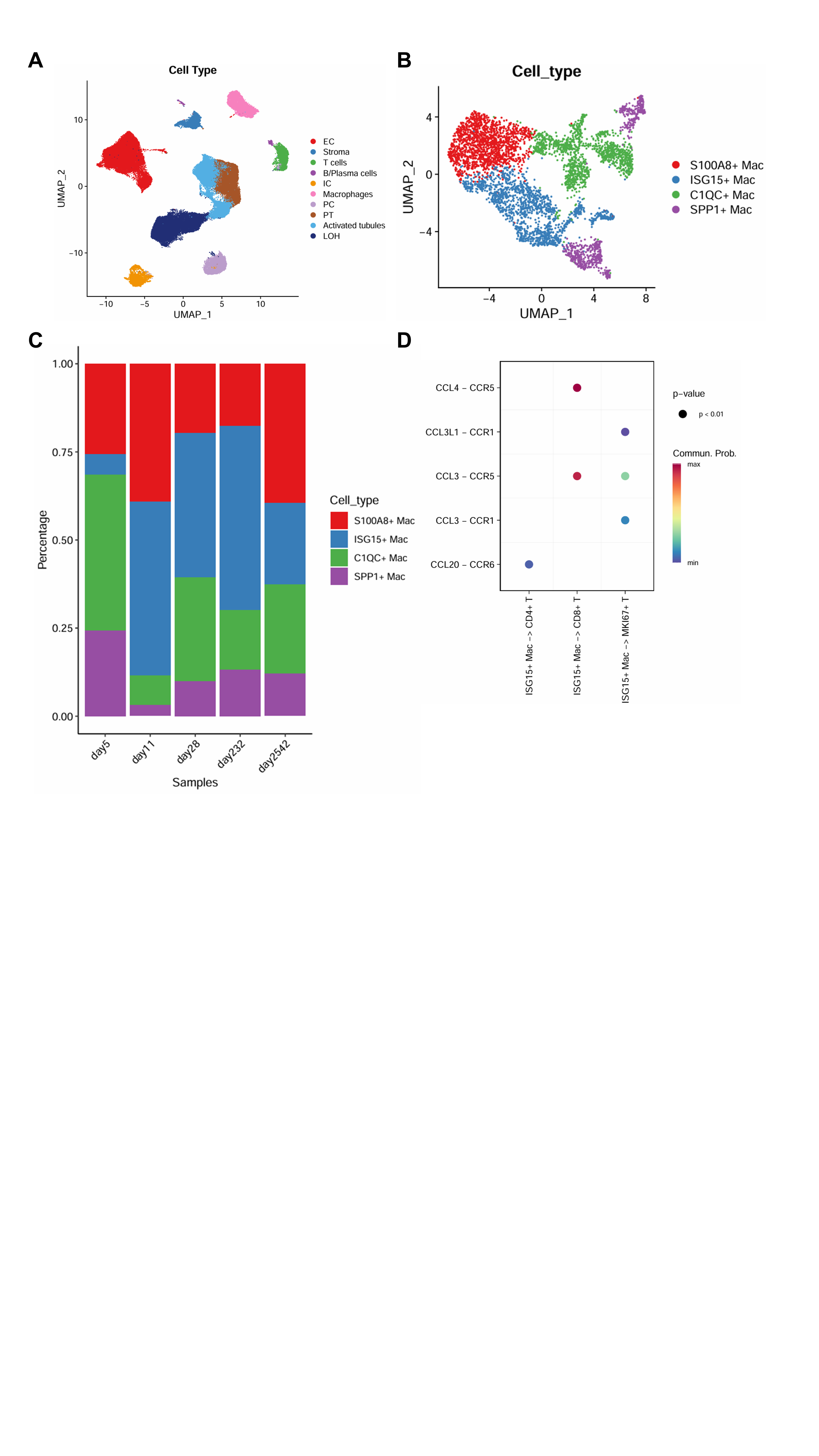

Supplement: Supplementary file 11 [file Image8.tif]

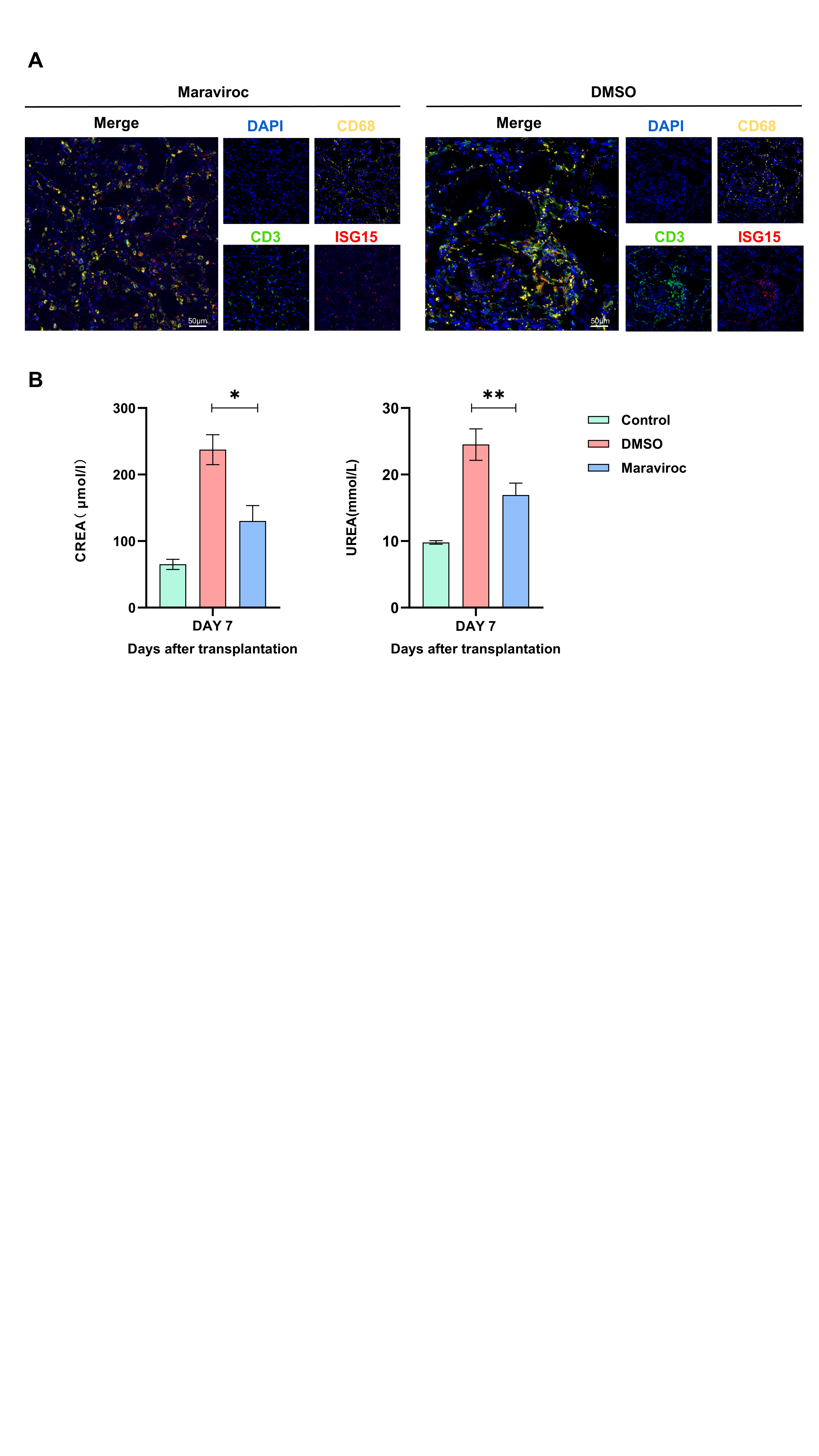

Supplement: Supplementary file 12 [file Image9.tif]
